# International consensus on terminology to be used in the field of echinococcoses

**DOI:** 10.1051/parasite/2020024

**Published:** 2020-06-03

**Authors:** Dominique A. Vuitton, Donald P. McManus, Michael T. Rogan, Thomas Romig, Bruno Gottstein, Ariel Naidich, Tuerhongjiang Tuxun, Hao Wen, Antonio Menezes da Silva, Dominique A. Vuitton, Donald P. McManus, Thomas Romig, Michael R. Rogan, Bruno Gottstein, Antonio Menezes da Silva, Hao Wen, Ariel Naidich, Tuerhongjiang Tuxun, Amza Avcioglu, Belgees Boufana, Christine Budke, Adriano Casulli, Esin Güven, Andreas Hillenbrand, Fateme Jalousian, Mohamed Habib Jemli, Jenny Knapp, Abdelkarim Laatamna, Samia Lahmar, Ariel Naidich, Michael T. Rogan, Seyed Mahmoud Sadjjadi, Julian Schmidberger, Manel Amri, Anne-Pauline Bellanger, Sara Benazzouz, Klaus Brehm, Andreas Hillenbrand, Fateme Jalousian, Malika Kachani, Moussa Labsi, Giovanna Masala, Antonio Menezes da Silva, Mahmoud Sadjjadi Seyed, Imene Soufli, Chafia Touil-Boukoffa, Junhua Wang, Eberhard Zeyhle, Tuerganaili Aji, Okan Akhan, Solange Bresson-Hadni, Chadli Dziri, Tilmann Gräter, Beate Grüner, Assia Haïf, Andreas Hillenbrand, Stéphane Koch, Michael T. Rogan, Francesca Tamarozzi, Tuerhongjiang Tuxun, Patrick Giraudoux, Paul Torgerson, Katherina Vizcaychipi, Ning Xiao, Nazmiye Altintas, Renyong Lin, Laurence Millon, Wenbao Zhang, Karima Achour, Haining Fan, Thomas Junghanss, Georges A. Mantion

**Affiliations:** 1 National French Reference Centre for Echinococcosis, University Bourgogne Franche-Comté and University Hospital FR-25030 Besançon France; 2 Molecular Parasitology Laboratory, Infectious Diseases Division, QIMR Berghofer Medical Research Institute AU-4006 Brisbane Queensland Australia; 3 Department of Biology and School of Environment & Life Sciences, University of Salford GB-M5 4WT Manchester United Kingdom; 4 Department of Parasitology, Hohenheim University DE-70599 Stuttgart Germany; 5 Institute of Parasitology, School of Medicine and Veterinary Medicine, University of Bern CH-3012 Bern Switzerland; 6 Department of Parasitology, National Institute of Infectious Diseases, ANLIS “Dr. Carlos G. Malbrán” AR-1281 Buenos Aires Argentina; 7 WHO Collaborating Centre for Prevention and Care Management of Echinococcosis and State Key Laboratory of Pathogenesis, Prevention and Treatment of High Incidence Diseases in Central Asia CN-830011 Urumqi PR China; 8 Past-President of the World Association of Echinococcosis, President of the College of General Surgery of the Portuguese Medical Association PT-1649-028 Lisbon Portugal

**Keywords:** Cystic Echinococcosis, Alveolar Echinococcosis, Neotropical Echinococcosis: *Echinococcus* spp., Terminology, Formalized consensus

## Abstract

Echinococcoses require the involvement of specialists from nearly all disciplines; standardization of the terminology used in the field is thus crucial. To harmonize echinococcosis terminology on sound scientific and linguistic grounds, the World Association of Echinococcosis launched a Formal Consensus process. Under the coordination of a Steering and Writing Group (SWG), a Consultation and Rating Group (CRG) had the main missions of (1) providing input on the list of terms drafted by the SWG, taking into account the available literature and the participants’ experience; and (2) providing independent rating on all debated terms submitted to vote. The mission of the Reading and Review Group (RRG) was to give an opinion about the recommendation paper in terms of readability, acceptability and applicability. The main achievements of this process were: (1) an update of the current nomenclature of *Echinococcus* spp.; (2) an agreement on three names of diseases due to *Echinococcus* spp.: Cystic Echinococcosis (CE), Alveolar Echinococcosis (AE) and Neotropical Echinococcosis (NE), and the exclusion of all other names; (3) an agreement on the restricted use of the adjective “hydatid” to refer to the cyst and fluid due to *E. granulosus sensu lato*; and (4) an agreement on a standardized description of the surgical operations for CE, according to the “Approach, cyst Opening, Resection, and Completeness” (AORC) framework. In addition, 95 “approved” and 60 “rejected” terms were listed. The recommendations provided in this paper will be applicable to scientific publications in English and communication with professionals. They will be used for translation into other languages spoken in endemic countries.

## Abstract (fr)

Les échinococcoses impliquent l’intervention de spécialistes de presque toutes les disciplines et une standardisation de la terminologie utilisée dans le domaine est donc cruciale. Pour harmoniser la terminologie des échinococcoses sur des bases scientifiques et linguistiques bien étayées, l’Association Mondiale de l’Échinococcose a entrepris un processus de « Consensus Formalisé ». Sous la coordination d’un Groupe de Pilotage et de Rédaction (GPR), un Groupe de Consultation et de Classement (GCC) a reçu les missions suivantes : (1) fournir un avis sur une liste de termes établie par le GPR, en prenant en compte les références scientifiques disponibles et l’expérience des participants ; (2) fournir un classement indépendant sur tous les termes débattus et soumis au vote. La mission du Groupe de Lecture et de Revue critique (GLR) était de donner un avis formel sur l’article de recommandations en termes de facilité de lecture, d’acceptabilité et d’applicabilité. Les principales avancées obtenues au terme de ce processus sont les suivantes: (1) une actualisation de la nomenclature actuelle des espèces d’*Echinococcus* ; (2) un accord sur les noms des trois principales maladies humaines dues aux espèces d’*Echinococcus* : l’échinococcose kystique (EK), l’échinococcose alvéolaire (EA) et l’échinococcose néotropicale (EN), à l’exclusion de toute autre dénomination ; (3) la restriction de l’usage de l’adjectif « hydatique » au kyste et au liquide/fluide produit par *E. granulosus sensu lato* ; et (4) une description standardisée des interventions chirurgicales pour l’EK, selon le système AORC (pour « Approche », « Ouverture », « Résection » et « Complétude »). De plus, 95 termes « approuvés » et 60 termes « rejetés » ont été listés. Les recommandations données dans cet article seront applicables aux publications scientifiques en anglais et à la communication avec les professionnels. Elles seront utilisées pour la traduction dans les autres langues parlées dans les zones d’endémie.

## Introduction

*Echinococcus* species (spp.) are parasites of the class Cestoda and belong to the phylum Platyhelminthes; they cause a variety of diseases in humans, most importantly cystic echinococcosis (CE; also found in scientific publications and professional/public communications under “hydatid cyst”, or “hydatid disease”, “hydatidosis”, “echinococcus cysticus”, etc.), alveolar echinococcosis (AE; also found under “alveococcosis”, “echinococcus alveolaris”, “alveolar hydatid”, “alveolar hydatidosis”, “alveolar hydatid disease”, “multilocular hydatid cyst”, “multilocular hydatid disease”, “multilocular hydatidosis”, etc.), and neotropical echinococcosis (NE; also found under “polycystic echinococcosis”, “polycystic hydatid disease”, “hydatidosis of the New World”, etc.) [35]. The simple enumeration of the various alternative names of the diseases, still in use in 2020, readily shows the absence of standardization of the terminology used in this field. Echinococcoses are zoonoses, and the complexity of the life cycle of the various species of *Echinococcus* involved, the variety of their hosts, as well as the impact of the diseases due to *Echinococcus* spp. on public health, require the involvement of specialists from nearly all disciplines (medicine and surgery, veterinary medicine, zoology, parasitology, ecology, agriculture, public health, economics, etc.) which have their own history and their own jargon. However, they must cooperate to solve the multiple problems of *Echinococcus* spp. infection, within the “One-Health” concept. This makes the use of a common vocabulary crucial. Time has thus come for standardization of the terminology in the field of echinococcosis.

From Hippocrates, who first described the cysts in patients more than 2400 years ago [18], to the 20th century, languages of Greek and Latin origin have served to name the parasites/diseases associated with *Echinococcus* spp. because in the Western world, countries around the Mediterranean were the known endemic regions for CE, the only recognized *Echinococcus*-related disease [13]. In the 19th century, physicians and researchers of German and Russian languages and in the 20th century, of French and English languages [18] brought new terms, and AE, then NE, were added to the list of *Echinococcus*-related diseases. At the end of the 20th century, China was recognized the main endemic region for CE and AE. Meanwhile, publishing in English became more and more common for physicians and scientists working in the field of echinococcosis worldwide, and everyone had to work with the variety of words and expressions describing *Echinococcus* spp. and echinococcosis in international publications in English [77]. Differences in wording, in the various professional environments, as well as in the various endemic countries, have become sources of misunderstanding, with influences related to the local language of the professionals.

The first decade of the 21st century, with the development of molecular biology techniques and the complete elucidation of the genomes of *E. granulosus* (Batsch, 1786) [87] and *E. multilocularis* (Leuckart, 1863) [73], has seen the emergence of new species within the genus *Echinococcus*, that have increased and refined our taxonomic knowledge of these parasites; such developments have also had an impact on several domains of echinococcosis research including not only genomics, proteomics and metabolomics, but also immunology and epidemiology [81, 86]. There has been and still is coexistence of already defined species based on genetic sequences with “types”, and “strains”, based on morphological characteristics [55]. A better definition of the limits of the species has led to an agreement between specialists on the definition and names of the “new” species, and nine species are now recognized and characterized by the sequences of their genomes, and defined in terms of host species and endemic areas [81]. The application of binomial nomenclature for genera and species is now governed by various internationally agreed codes of rules, of which the “International Code of Zoological Nomenclature (ICZN)” governs the scientific designation of parasites (https://www.iczn.org/the-code/the-international-code-of-zoological-nomenclature/the-code-online/). This international system must be followed, and any new species designation has to be registered for its international use. However, the translation of the “new” *Echinococcus* species defined by specialists into common usage by professionals has not yet been achieved, and many publications still use “*E. granulosus*” as a single species name responsible for CE.

The situation is also problematic for disease names and all other terms and expressions. This may be due to the status of echinococcoses as “orphan” diseases or “neglected diseases” (e.g., for the World Health Organization they are now recognized as “Neglected Tropical Diseases” (https://www.who.int/neglected_diseases/diseases/en/), even though most human cases and animal infections are not in tropical areas). For “non-neglected” diseases, authoritative international scientific societies have fixed the terminology and published recommendations that are regularly updated. The World Federation of Parasitologists has endorsed the Standardised Nomenclature of Parasitic Diseases (SNOPAD), initially published in 1988 for animal parasitic diseases by the World Association for the Advancement of Veterinary Parasitology, and has established rules for the names of parasitic diseases [32] (cf. https://www.waavp.org/documents/snopad-guidelines/#.XS9gBvIza00). The disease names are derived from the genus of the parasite with a suffix in “-osis” (e.g., *Echinococcus* would give “echinococcosis”). The rule is simple and clear; but the application of this rule has not been consistently followed and, especially for echinococcosis, coexistence of various names prevails, with a subsequent impact on database searching and misunderstanding between researchers and professionals [33, 34]. Other terms used in the “echinococcosis field” are equally ill-defined.

The “*Asociación Internacional de Hidatidología*” was founded in 1941 in Colonia del Sacramento (Uruguay), during the first “International South American Conference to Fight against Hydatidosis”, aiming at coordinating echinococcosis control, especially in the endemic countries of South America, and organizing conferences. From 1951 to the end of the 1990s, Spanish, French and English were the official languages of the biennial “World Congress of Hydatidology”, the main activity of the “International Association of Hydatidology” (IAE). At the 26th congress in Bucharest, Romania, in 2015, the name of the association in English was officially changed to “World Association of Echinococcosis (WAE)”, a formal step towards the adoption of “echinococcosis” for the denomination of the diseases (and of the field of research in general). In 1985, the WHO Informal Working Group on Echinococcosis (IWGE) was created, with the double aim of creating a network of scientists working in basic sciences in the realm of echinococcosis, and in standardizing practices for the diagnosis and treatment of *Echinococcus* spp. – related diseases, in line with the priorities of the WHO [18].The group is currently working on a Technical Manual to help clinicians in the care management of patients with echinococcosis. Both professional entities (IAE and IWGE) hold their main meeting at the same location and have always joined efforts to issue manuals on echinococcosis including veterinary aspects, prevention and control [15–17], or write and update guidelines for diagnosis and treatment of patients with echinococcosis [10, 83]. In a plenary session of the 27th World Congress of Echinococcosis (WCE) in Algiers, Algeria, 2017, the need for harmonizing the terminology of echinococcosis, on sound scientific and linguistic grounds, was stressed; a working group and a consensus process were established to provide recommendations applicable to scientific publications in English, and to the communication between professionals. Such recommendations should then be used as a basis for translation into other languages spoken in endemic countries for communication, teaching and training. This report and its and its illustrations (Tables and Figures) and Appendix are the ultimate result of the process.

## Materials and methods

The methodology proposed to the participants in the working groups established at the 27th WCE was the “Formal consensus” approach, as used by the French “*Haute Autorité de Santé*” (https://www.has-sante.fr/upload/docs/application/pdf/2018-03/good_practice_guidelines_fc_method.pdf), inspired by both the Delphi process [30] and the Consensus Conference methods [19]. As the methodology was initially described to set up guidelines for good clinical practices, the method was slightly modified, as described below, to fit with the objectives of the present work, i.e. an agreement on terminology and good publishing practices. All participants in the various groups involved in the “formal consensus” process are cited as “associated authors” in the final publication (names in Appendix).

### Definition and modalities of constitution of the Working Groups

#### Steering and Writing Group (SWG)

The mission of the SWG was: (1) to draft the list of words and expressions, and provide a critical analysis of their scientific and linguistic relevance, from the international scientific literature and all available sources (international nomenclature, international recommendations, historical notes, etymology), and submit it to the participants in the Consultation and Rating Group (CRG); (2) to collect and discuss the feedback from the participants in the CRG, and if necessary to submit difficult cases to appropriate external experts; (3) to draft the final lists of words and expressions and submit them to rating by the participants in the CRG; (4) to select 12 experts in the various disciplines of interest to the field of echinococcosis, not involved in the CRG, as members of the Reading and Review Group (RRG); (5) to analyze the rating data and draft the initial version of the Recommendation Paper, including the final tables with recommended terminology, to be submitted to the RRG; (6) to consider the remarks of the RRG on the draft, finalize the paper and the tables with the members of the CRG, and submit it to the appropriate journal; and (7) to ensure the follow-up of the paper and the dissemination of its content to end-users.

The composition of the SWG, chaired by a project manager, was approved by the president and the members of the WAE attending the 27th WCE, and three topics of interest were proposed, each coordinated by two members of the SWG, experts in the area.

#### Consultation and Rating group (CRG)

The mission of the CRG in the “formal consensus” process was (1) in the first step of consultation, to provide input on the initial list of terms and expressions drafted by the SWG, taking into account the available literature in all fields and the personal experience of the various participants; (2) in the second step of consultation, to participate in the poll, and provide independent rating on all words and expressions submitted to vote by the SWG; and (3) to finalize the text of the paper with the SWG after its evaluation by the RRG, and before its submission to the appropriate journal.

Sub-groups, corresponding to the three topics of interest were composed on a voluntary basis at the 27th WCE, and completed after a call for volunteering launched by email by the SWG after the congress. On their request, participants could belong to more than one subgroup. The list of the CRG participants who were involved in the whole process, and their country/countries of residence and field work, is provided in the Appendix. For the “Species and epidemiology”, “Biology and immunology” and “Clinical aspects” subgroups of the CRG, 17, 18, and 14 participants were identified, respectively from 15 countries in total. The project manager coordinated all activities of the groups but was not involved in any rating; however, other members of the SWG who volunteered for the task could be involved in rating but only for those subgroups they were not coordinating.

#### Reading and Review Group (RRG)

The mission of the RRG, multidisciplinary and multi-professional, was to give a formal opinion on the content and form of the initial version of the paper and the recommendations it may contain, especially in terms of applicability, acceptability, and readability in their own domain. According to the rules of the “formal consensus” process, the participants in the RRG would offer an advisory opinion on an individual basis, without *de novo* questioning the decisions from the votes obtained in the first 2 steps of the process. The list of the RRG participants (four experts for each topic) who were involved in the reviewing process, and their country/countries of residence and field of expertise, is given in the Appendix.

### Schedule of the process

**Figure 1:**
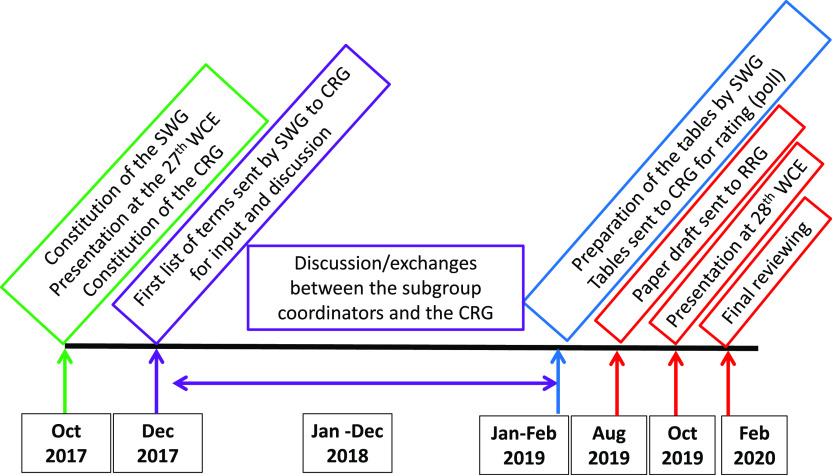
Time schedule and main activities within the “Formal consensus” process for the definition of the terminology of echinococcosis.

The time schedule of the process, from October 2017 to February 2020, is given in Figure 1. The main steps included: (1) constitution of the SWG, presentation of the project, and constitution of the CRG; (2) provision of a list of words and expressions, with arguments for their acceptance or rejection, provided by the SWG to the participants in the CRG; (3) input from the participants in the CRG and open discussion; (4) constitution of the RRG by the SWG; (5) a preliminary summary by the SWG, and preparation of three lists of words and expressions for each of the three topics, according to three levels of decision: (a) *a priori* approved terms, (b) *a priori* rejected terms, (c) debated issues submitted to vote; (6) vote on “debated issues” by the participants in the CRG; (7) analysis of data and drafting of the Recommendation Paper by the SWG; (8) reviewing of the draft by the RRG; (9) last review by the CRG; (10) submission of the manuscript by the SWG; and (11) follow-up of the publication process and dissemination of the recommendations by the SWG.

Except for the first kick-off meeting at the 27th WCE and the final presentation at the 28th WCE in Lima, Peru, and given the worldwide extent of the consultation group and absence of funding, there were no further face-to-face meetings; all discussions and exchanges were managed by email. The project manager and members of the SWG answered all messages sent by the members of the CRG and all contributions were taken into account at any stage.

### Analysis of the poll

Medians and means of the ratings, on a possible agreement range from 1 to 10, were calculated for the answers to each question. A majority of agreement for approval or rejection was considered when more than half of the ratings were equal to or higher than 5, or equal to or lower than 5, respectively. Unanimous approval or rejection was defined as ratings higher than or lower than 5 for all voters, respectively. Agreement for rejection was defined for a median equal to 3.5 or lower, and majority or unanimity of ratings; agreement for approval was defined for a median equal to 7.5 and higher; and majority or unanimity of ratings. A question was deemed “undecided” when the median was between 3.5 and 7.5 and/or there was no majority of ratings. After the poll and when needed, i.e. whenever the result of the votes was “undecided” and/or whenever there was no full agreement on the definition of a term, the SWG submitted the term/expression to further discussion between specialists in order to reach final agreement. To this purpose, subgroups of specialists (e.g., surgeons) were constituted to propose final agreement.

## Results

### Final composition of the CRG subgroups

For the “Species and epidemiology” subgroup, 1 participant could not be reached by email and 1 never answered emails; the final rating was thus provided independently by 15 participants, from 10 countries. For the “Biology and immunology” subgroup, three participants could not be reached; five participants from the same research team worked together for the final rating; thus the final rating was provided independently by 11/15 participants from 11 countries. For the “Clinical aspects” subgroup, one participant could not be reached and one did not answer emails; 2 participants from the same research group worked together for the final rating; thus the final rating was provided by 11/12 participants from 8 countries.

### Results of the first stage of consultation

The first stage of consultation generated numerous comments with associated arguments and references from all participants in the CRG. This input was carefully collated in a summary version of the preliminary tables by the SWG. In case of need, other experts of the specific field were consulted by the coordinators of the subgroups, and their opinion was brought to the attention of the CRG participants. The synthetic tables as well as additional pictures if necessary (especially for the subgroup on “Biology and immunology”), were again sent to all participants to obtain initial evaluation of possible consensus, and set up a list of still “debated issues”.

For the “Species and epidemiology” subgroup, it was agreed that the nomenclature of old and new species of *Echinococcus* should follow the rules of the ICZN. Thus, the names of the species (and their number) listed in the tables reflect the results of current taxonomic research and do not preclude future changes, provided these are based on convincing new evidence. There was also a consensus on the use of the “G” genotypes in cases where genetic differences are apparent, but where – based on current knowledge – these differences are deemed too small to warrant recognition of the variants as named taxa. In addition, the term “genotype” should be reserved for characterization using molecular biology/sequencing, and “strain” or “type” should be reserved for characterization using morphological and host-range characteristics. Consequently, there were no “debated issues” for this subgroup. However, definitions and clarifications on species names and on other words and expressions regarding this topic were given in provisional tables; they were submitted for approval or rejection in the second stage of the process (with an answer of the “YES/NO” type).

For the “Biology and immunology” subgroup, there was far more debate since there is no official institution in charge of such terminology. Divergences appeared about the various stages of development of the *Echinococcus* spp. in their intermediate hosts, and particularly on the definition of the term metacestode. Other minor divergences were noted on the use of certain words to designate the cells and components of these parasites, either adult or larval, as well as the host’s reaction. References were provided by parasitology and immunology specialists to support data interpretation, thus the use of specific words. From that stage of the consultation, there was general agreement that the adjective “hydatid” should only be used for *E. granulosus sensu lato*; as this was a sensitive issue, it was however kept in the “debated issues” submitted to vote, both for the “Biology and immunology” and for the “Clinical aspects” subgroups. For details on the names of the various components of the egg of all *Echinococcus* spp. and of the cyst in CE, it was decided to provide a figure that would fix the definitions of the various words (Figs. 2A, 2B and 2C). The definition of “cyst” was initially the main disagreement between “parasitologists” (2-layer cyst) and “clinicians” (3-layer cyst). At the end of the first stage, it was finally agreed by all experts, whatever their field of expertise, that all cysts due to *Echinococcus* spp. and, especially for clinicians, those due to *E. granulosus s.l*., included two “layers” (and not “membranes”) of parasite origin and one layer of host origin, thus three layers. This clarification helped when defining several derived terms.

**Figure 2A and B:**
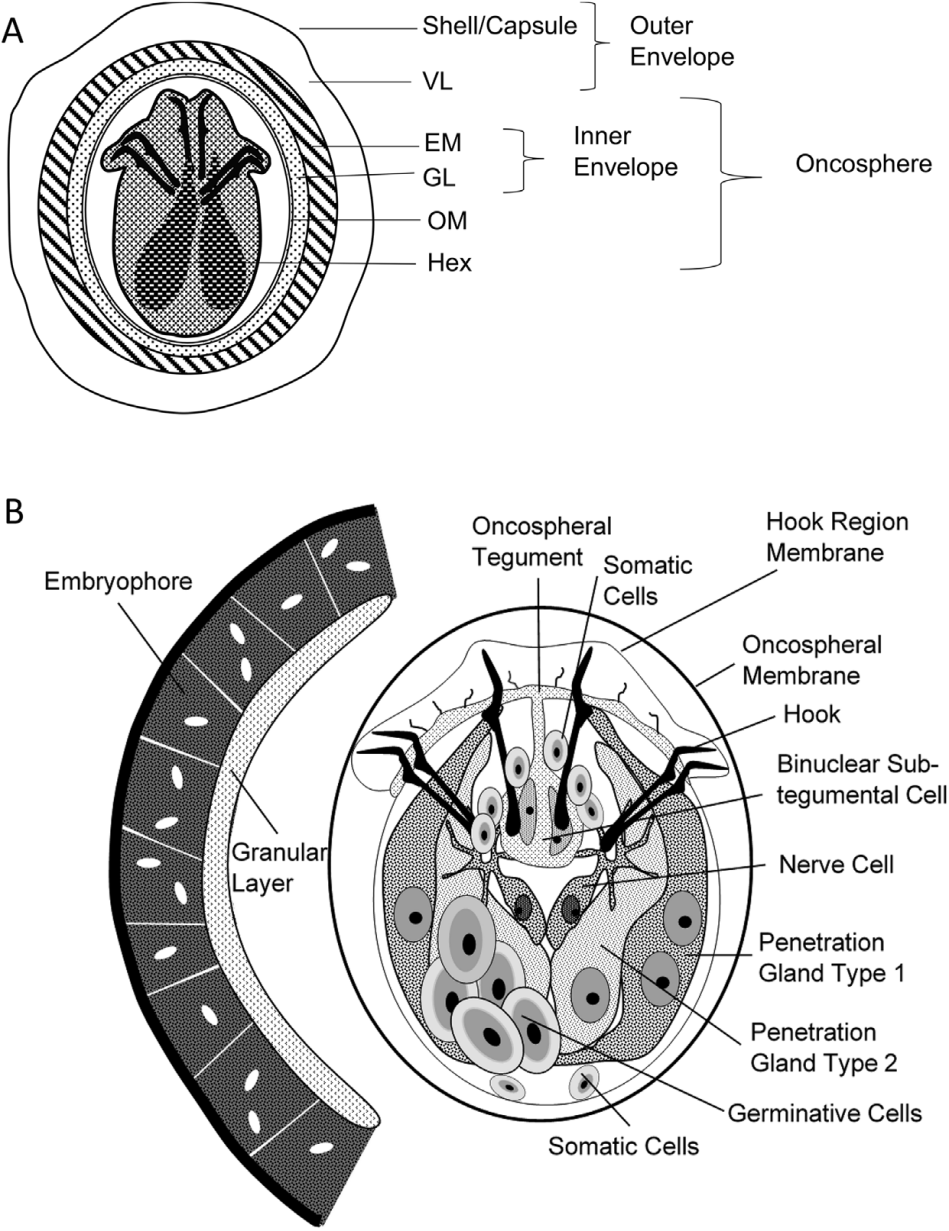
General description of the egg and oncosphere of *Echinococcus* spp, according to Jabbar et al., 2010 [27]. (A) Schematic diagram of an oncosphere illustrating the structure and bilateral symmetry in the pattern of hooks and cellular organization of the hexacanth embryo. VL: vitelline layer; EM: embryophore; GL: granular layer; OM: oncospheral membrane; Hex: hexacanth embryo. (B) Cellular organization of the oncosphere. Oncospheres are approximately 25 × 30 μm.

**Figure 2C:**
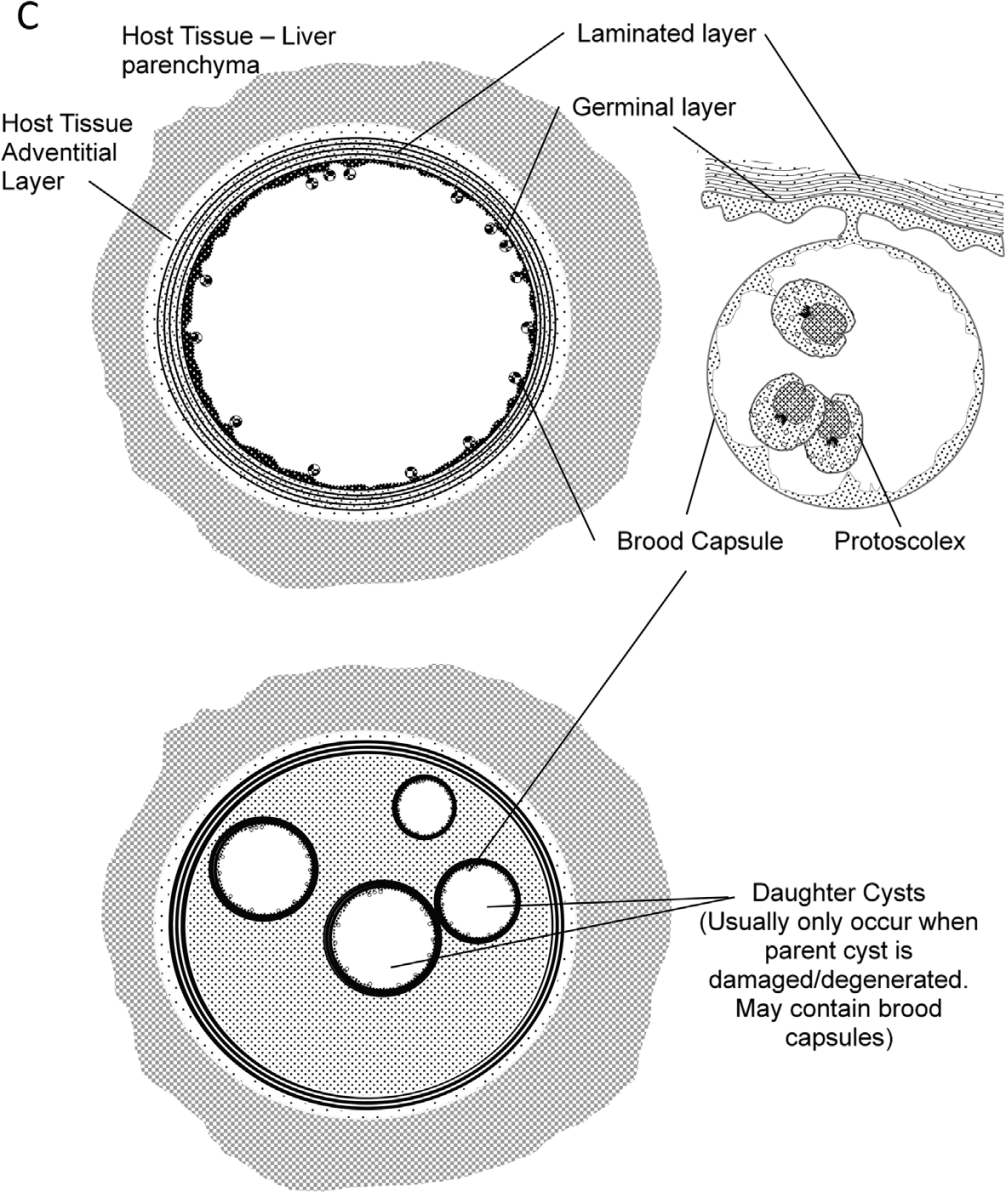
General description of the metacestode of *Echinococcus* spp. (see also Table 1B and Fig. 3).

For the “Clinical aspects” subgroup, it was readily agreed that the names of diseases due to the various species of *Echinococcus* should follow the rules proposed by the SNOPAD and the generic name “echinococcosis” should always be used, irrespective of the species and type of disease. “Cystic” and “Alveolar” should be used as adjectives to designate the diseases due to *E. granulosus* (Batsch, 1786) *sensu lato*, and to *E. multilocularis* (Leuckart, 1963), respectively. Some discussion remained for the use of the adjective “hydatid”, and for the names of the diseases due to *E. oligarthra* (Diesing, 1863) and *E. vogeli* Rausch and Bernstein, 1972, in South America. Most of the debated issues concerned the names of therapeutic interventions (surgical or using other techniques); however, the common definition of “cyst”, shared by parasitologists and clinicians, led to an easier consensus on the meaning of “cystectomy”.

### Results of the second stage of consultation

#### Agreement for approval or rejection

A few terms proposed in the lists for “approval” or “rejection” at the end of the first stage of the process still received objections from participants in the CRG. More precision (or references) were provided in order to reach a final consensus on these terms and their definition. Approved terms were provisionally included in Tables 1A (Genetics and epidemiology), 1B (Biology and immunology) and 1C (Clinical aspects). Rejected terms were provisionally included in Tables 2A (Genetics and epidemiology), 2B (Biology and immunology) and 2C (Clinical aspects).

**Table 1A:** Recommended terms for the genetics and epidemiology of *Echinococcus* species.

Word/expression	Definition	Arguments for acceptance, references, linguistic clarifications	Comments
*Echinococcus* Rudolphi, 1801 (Cestoda: Taeniidae)	Genus in the family Taeniidae Ludwig, 1886, order Cyclophyllidea, class Cestoda, phylum Platyhelminthes, kingdom Animalia.	International nomenclature; name of the genus *Echinococcus* [67]; must be written in *italics* with first letter in capital letter; abbreviation “*E.*” (italics followed by a dot) when in a binomen (i.e. a generic name and a specific name) or in a trinomen (a generic name, a specific name and a subspecific name – this is rare).	The genus name *Alveococcus* (for the species *Alveococcus multilocularis* (Leuckart, 1863) Abuladze, 1959) was erected to separate *E. multilocularis* from the other species. There is no longer a taxonomic basis for such a separation, but the name is still used occasionally, particularly in Russian literature. See also Table 2A.
*Echinococcus* sp.	One species within the genus *Echinococcus.*	The abbreviation “sp.” (not italics) stands for a species, the identity of which is not known (e.g. in case of an undetermined *Echinococcus* isolate)	
*Echinococcus* spp.	More than one (or all) species within the genus *Echinococcus.*	The abbreviation “spp.” does not indicate a species with the taxonomic definition of “species”, but stands for “*species pluralis*” (the Latin for “multiple species”), hence the roman letters (not italics).	
		spp is followed by a dot (because it is the abbreviation of a Latin expression; see e.g., i.e., as examples), thus: “spp.”	
*Echinococcus canadensis* (Webster & Cameron, 1961)	A species within the *E. granulosus sensu lato* species cluster.	*E. canadensis* (Webster & Cameron, 1961) [80] corresponds to the previous “G6/G7”, “G8” and “G10” genotypes, identified by DNA sequencing [52] (see GenBank: https://www.ncbi.nlm.nih.gov/genbank/); *E. canadensis* belongs to *E. granulosus s.l.*	*E. canadensis* cycle may involve camels and goats (G6 genotype), pigs (G7) and cervids (G8 and G10) as intermediate hosts and dogs (as well as wolves for G8 and G10) as definitive hosts; however, sheep, cattle and other ungulates may also be infected by *E. canadensis,* which is the second species within *E. granulosus s.l.,* after *E. granulosus s.s.,* to infect humans.
		Genotypes may be used to differentiate between distinct molecular sequences, within the defined species; the word “strain” should no longer be used.	G6 and G7 may in future be separated from this species, so at present the expression “*E. canadensis* cluster” is recommended.
*Echinococcus equinus* (Williams & Sweatman, 1963)	A species within the *E. granulosus sensu lato* species cluster.	*E. equinus* (Williams & Sweatman, 1963) [84] corresponds to the previous “G4” genotypes, identified by DNA sequencing [52] (see GenBank: https://www.ncbi.nlm.nih.gov/genbank/). *E. equinus* belongs to *E. granulosus s.l.*[66]	*E. equinus* cycle usually involves members of the horse family as intermediate hosts and dogs as definitive hosts. It is also known from a wildlife cycle involving lions and zebras [9, 78].
			It is associated with disease in non-human primates (lemurs) [9, 12].
			However, the zoonotic potential of *E. equinus* infection (i.e. infection in humans) has not yet been convincingly demonstrated [2].
*Echinococcus felidis* Ortlepp, 1937	A species within the *E. granulosus sensu lato* species cluster.	*E. felidis* Ortlepp, 1937 [59], was first described as such in 1937 by Ortlepp from the lion*, Panthera leo,* in South Africa; it is now recognized as a distinct species, identified by DNA sequencing [52] (see GenBank: https://www.ncbi.nlm.nih.gov/genbank/collab/); *E. felidis* belongs to *E. granulosus s.l.* [26]	*E. felidis* cycle involves lions as definitive hosts, and it is only known from warthogs and hippos as intermediate hosts; *E. felidis* infection has not been recognized to be associated with disease in humans up to now [25].
*Echinococcus granulosus* (Batsch, 1786) *sensu lato*	The concept of *E. granulosus* as one species cluster that contains all agents of cystic echinococcosis.	“*sensu lato*” and “*sensu stricto*” are used, when a species name (here: *E. granulosus* (Batsch, 1786)) [7] is used in different concepts: in a wider sense (*s.l*.) that includes e.g. cryptic species, or in a more restricted concept (*s.s*.) [52].	“*granulosus*” should be followed by “*sensu lato*” whenever the precise species has not been determined.
		“*sensu lato*” should be in italics (although this is debated), without capital letters as first letters. The abbreviation of each word in “*sensu lato*” is followed by a dot (because it is the abbreviation of Latin words; see e.g., i.e., as examples), thus: “*s.l.*”	For publications on the clinical aspects of echinococcosis, as the clinical descriptions of the disease “cystic echinococcosis”(CE) fits with all those species within *E. granulosus sensu lato* that are responsible for disease in humans, the use of *E. granulosus sensu lato* is allowed when there was no identification of the real species.
			For publications on basic research and epidemiology, molecular identification of the real species is necessary; it should be performed and indicated in the Materials and Methods section.
*Echinococcus granulosus* (Batsch, 1786) *sensu stricto*	A species within the *E. granulosus sensu lato* species cluster.	“*sensu stricto*” and “*sensu lato*” are used, when a species name (here: *E. granulosus* (Batsch, 1786)) [7] is used in different concepts: in a wider sense (*s.l*.) that includes e.g. cryptic species, or in a more restricted concept (*s.s*.) [52, 66].	*E. granulosus sensu stricto* corresponds to the previous “G1”, “G2”, a microvariant of “G3”, and “G3” genotypes, identified by DNA sequencing (see GenBank: https://www.ncbi.nlm.nih.gov/genbank/); its cycle usually involves sheep as intermediate hosts and dogs as definitive hosts; cattle and other ungulates may also be infected by *E. granulosus sensu stricto*
		“*sensu stricto*”, which is the equivalent of a species, should be in italics (although this is debated), without capital letters as first letters. The abbreviation of each word in “*sensu stricto*” is followed by a dot (because it is the abbreviations of Latin words; see e.g., i.e., as examples), thus: “*s.s.*” *E. granulosus s.s.* belongs to *E. granulosus s.l.* [66]	A strongly diverging genotype from Africa (“G Omo”) is provisionally retained in *E. granulosus s.s*., but will have to be reclassified in the future [79].
*Echinococcus multilocularis* (Leuckart, 1863)	A species in the genus *Echinococcus.*	*E. multilocularis* (Leuckart, 1863) [50], is the agent of the disease “alveolar echinococcosis” (AE) in humans [35, 52]	*E. multilocularis* was clearly distinguished from *E. granulosus sensu lato* in the middle of the 20th century [18, 77]. It involves a variety of rodents and lagomorphs as intermediate hosts depending on the geographical area, humans as accidental intermediate hosts, and various species of foxes, dog and wolf as definitive hosts. The disease caused by *E. multilocularis*, characterized by lesions composed of an aggregate of microcysts embedded in a granulomatous host’s reaction, is distinct from that caused by *E. granulosus s.l.* as well as those caused by *E. vogeli* and *E. oligarthra*.
			Since then, no major genetic polymorphism has been found within *E. multilocularis* that would distinguish new species or even strains. For phylogeographic studies (variability at a continental scale), genetic polymorphism within the micro-satellite EmSB may be used [42].
*Echinococcus oligarthra* (Diesing, 1863)	A species in the genus *Echinococcus.*	*E. oligarthra* (Diesing, 1863) [14] is a species found in South, Central and North America (Mexico) [52, 58].	*E. oligarthra* cycle usually involves agouti (*Dasyprocta* spp.) and occasionally paca (*Cuniculus paca*), spiny rats (*Proechimys* spp.), rabbits (*Sylvilagus floridanus*), and opossums (*Didelphis marsupialis*) as intermediate hosts and various wild cat species as definitive hosts [65].
		The component “arthra”, originally proposed by Diesing, comes from the ancient Greek ἄρθρα –arthra (joints) which is the plural of ἄρθρον -arthron (joint). The name is therefore not an adjective, but a noun in apposition, which does not change its ending according to the gender of the generic name. This was recognized earlier but subsequently ignored.	It is responsible for a disease in humans distinct from cystic and alveolar echinococcosis, sometimes wrongly called “polycystic echinococcosis” (since it usually presents as a single cyst) [35].
			“Neotropical echinococcosis” is the expression recommended to qualify human infection due either to *E. vogeli* or to *E. oligarthra.* See also Table 1C
*E. ortleppi* Lopez-Neyra & Soler Planas, 1943	A species within the *E. granulosus sensu lato* species cluster.	*E. ortleppi* Lopez-Neyra & Soler Planas, 1943 [51] corresponds to the previous “G5” genotypes, identified by DNA sequencing [1, 52, 66] (see GenBank)	*E. ortleppi* cycle usually involves cattle as intermediate hosts and dogs as definitive hosts; other ungulates may also be infected by *E. ortleppi* [24]*.* Human cases are known, but rare [13, 24].
*E. shiquicus* Xiao, Qiu, Nakao, Li, Yang, Chen, Schantz, Craig & Ito, 2005	A species in the genus *Echinococcus.*	*E. shiquicus* Xiao et al., 2005 [85] is a species phylogenetically close to *E. multilocularis* [52] which was identified in the county of Sêrxü (in Tibetan, 石渠县 in Mandarin Chinese (pinyin: Shíqú Xiàn)) in Sichuan province, Qinghai-Tibet plateau region of Western PR China	*E. shiquicus* (pronunciation “seshüicus”) infection has not been recognized to be associated with disease in humans up to now [35].
*E. vogeli* Rausch & Bernstein, 1972	A species in the genus *Echinococcus.*	*E. vogeli* Rausch & Bernstein, 1972 [62] is an *Echinococcus* species found only in South and Central America.	*E. vogeli* natural cycle mainly involves paca (*Cuniculus paca*) as intermediate hosts; it has also been documented in other rodents such as agouti (*Dasyprocta spp.*)*,* and the bush dog (*Speothos venaticus*) and domestic dogs as definitive hosts [65]; *E. vogeli* is responsible for a disease in humans distinct from cystic and alveolar echinococcosis, often called “polycystic echinococcosis”, because of the presentation of the disease [35].
			“Neotropical echinococcosis” is the expression recommended to qualify the human infection due either to *E. vogeli* or to *E. oligarthra*: see also Table 1C.
G genotypes	Genotypes identified within the species *E. granulosus s.l.,* previously identified as “strains”.	Genotypes may be used to differentiate between distinct molecular sequences, within the defined species or complexes; the word “strain” should no longer be used when genetic characterization has been performed [52, 55].	Further definition of new species is still possible. Waiting for such definitions, G genotypes should be kept, in addition to the current species name, if necessary.
G1 and G3 genotypes	Genotypes individualized within *E. granulosus sensu stricto.*	The word “genotype” should be used; it is more appropriate than “strain” since it is based on genotyping and not on other techniques (especially morphological) previously used to distinguish between strains [52, 55].	Keeping the distinction between genotypes may be necessary for phylogeographic studies and in some circumstances for echinococcosis control (targeting animal hosts and specific cycles) [38, 41]. Best target for identification of G1 and G3 seems to be the *nad5* gene region [39]. G1 is the most common genotype in sheep; G2 was first described as “Tasmanian sheep strain” but is actually cosmopolitan; G3 was initially identified from water buffalo, but is now also known from other intermediate hosts.G2 is considered a microvariant of G3 [40].
G6/G7 G8 G10	Genotypes individualized within *E. canadensis* (Webster & Cameron, 1961).	Pending further distinction between accepted species, genotypes within *E. canadensis* should be qualified by the previously accepted “G” numbers G6/G7, G8 and G10 [49, 66].	G9 is no longer recognized as a distinct genotype; it is probably a microvariant of G7; the genotypic cluster G6/7 is the second most important agent of human CE worldwide; further distinction between species within the *E. canadensis* cluster has been proposed, but has not been accepted yet [48, 53, 54, 57].
		Genotypes may be used to differentiate between distinct molecular sequences, within the defined species; the word “strain” should no longer be used.	

**Table 1B:** Recommended terms for the biology and immunology of *Echinococcus* species.

Word/expression	Definition	Arguments for acceptance; references; linguistic clarifications	Comments
* **Aborted (cyst, lesion),** Adjective	Non-viable parasitic structure as evidenced by imaging (complete calcification in AE, CE5 cyst in CE) or histological examination (absence of viable parasitic cells).	Could be used in clinical or experimental situations when evidence of absence of viability is not obtained by transplantation or *in vitro* culture.	Distinct from “non-viable”; the definition was approved unanimously by the voters; the adjective “aborted” was preferred to the alternative “died-out” by the majority of voters (median: 10).
* **Adult form,** Expression	Sexual reproduction stage of *Echinococcus* spp. parasites in their definitive hosts.	More popular alternative expression to the specific scientific expression “strobilar stage” to designate this step in the development of *Echinococcus* spp. cestodes; expression coherent with “larval form” in the intermediate hosts.	“Adult” may include all stages of development in the definitive host (fertile or immature worms i.e. with the last segment containing eggs or not).
			This expression with its definition was approved by the majority of voters (median: 10)
* **Adventitial layer,** Expression	Partly cellular and fibrous layer of host origin surrounding the inner 2 layers of parasite origin (germinal and laminated layers) of the metacestode in the intermediate host of *E. granulosus sensu lato*	The expression was approved in order to have a coherent denomination of the various “layers” of the hydatid cyst, irrespective of their origin (parasite or host).	“Adventitia” was an alternative term; it was rejected by voters (median: 2). The expression “adventitial layer” with its definition was approved by the majority of voters (median: 10)
		As for “germinal membrane” and “laminated membrane”, the use of the expression “adventitial membrane” is not relevant.	The correct description of the parasitic structures in the intermediate host thus includes 3 similar expressions, from inside to outside: Germinal layer Laminated layer Adventitial layer
* **Border (periparasitic),** Noun	For both *E. granulosus s.l.* and *E. multilocularis*, structures that surround the laminated layer, at the border with the organ parenchyma.	Alternative term to “boundary”	The use of “boundary” was rejected by all voters but one.
			The noun “border” with its definition was approved by the majority of voters (median: 10).
**Brood capsule,** Expression	Element budding from the germinal layer which produces protoscoleces.	No synonyms.	In some publications, some confusion may be found with “daughter cysts”. The 2 entities are totally different in their origin and their components (see Figs. 2A–2C).
* **Cyst (echinococcal or hydatid),** Noun	Anatomical entity produced by the growth of the metacestode of *Echinococcus* spp. distinct from the surrounding organ parenchyma and filled with fluid, which includes (from outside to inside) Adventitial layer (of host origin, even reduced to a few infiltrating cells or to fibrous tissue) Laminated layer (of parasite origin) Germinal layer (of parasite origin) - Cyst fluid (and its content, of both parasite and host origin).	The word “cyst” should be reserved to the clinical (or experimental) situation in CE (“hydatid” cyst) or NE; it may be observed by a variety of imaging techniques such as ultrasound imaging, computed tomography, or magnetic resonance imaging.	Should never be used to designate the central necrotic cavity often developed in the AE lesions. “Pseudocyst” should be used for this cavity.
		The international classification of CE cysts is based on ultrasound imaging with 5 CE stages [82]	All voters supported the restriction of the noun “cyst” to CE or NE, in the context of echinococcosis (median: 10).
		Two types of CE3 cysts (a and b) are now described [10, 31].	
* **Cyst fluid,** Expression	Liquid secreted by the germinal layer of *Echinococcus* spp. larval form and collected at the center of the “cyst” (for *E. granulosus s.l.*) or of the “microcysts” (for *E. multilocularis*).	Should be reserved for *in vivo* situations: hydatid cysts in CE, and NE, and microcysts of AE in human and animal hosts, including experimental models.	The distinction between “vesicle fluid” and “cyst fluid” was approved by all voters (median: 10); they also approved the use of “cyst” for the *in vivo* situation only.
* **Echinococcal,** Adjective	Adjective proposed to qualify anything relating to all *Echinococcus* spp. irrespective of the species, of the pathology, or of the clinical presentation of the disease that occurs in humans or in animal intermediate or definitive hosts.	Completely generic term that does not assume the species and/or stage of *Echinococcus* spp.	The term “hydatid” is more common, but also more confusing, and should be restricted to *E. granulosus s. l.* Usage of “echinococcal” with the generic definition was approved by the majority of voters (median: 8).
		Based on the recommendations of the World Federation of Parasitologists regarding the names of parasitic diseases [32].	
		https://www.waavp.org/documents/snopad-guidelines/#.XS9gBvIza00	
**Egg,** Noun	Product of the hermaphrodite fecundation in the last proglottis of the adult form of *Echinococcus* spp. parasites; released in the feces of the definitive hosts and the environment.	Should be restricted to the stages preceding ingestion by the intermediate host and oncosphere release [27].	See Figures 2A–2C for detailed description and terminology.
**Fertile (strobilar stage/adult form, or worm)**, Adjective	Adult form/strobilar stage (worm) of *Echinococcus* spp. in the definitive host, the last segment of which contains eggs (i.e. infectious to intermediate hosts).	Production of eggs, observable in the last segment of the adult form implies that the worm is fertile (conversely, absence of eggs does not automatically imply that it is non-fertile; it may just be “immature”)	Please see also the definition of “fertile” for the parasitic structures in the intermediate host, and the definition of “immature”, in this table.
* **Fertile (metacestode, larva, cyst, microcyst…),** Adjective	Larval parasitic structure that contains viable protoscoleces and thus allows infection of the definitive hosts through scoleces or in some pathological or experimental circumstances production of newly developed metacestodes in the intermediate host.	Production of protoscoleces by the germinal layer and their release in the parasitic fluid (whatever the species) implies that the metacestode is fertile.	This adjective with its definition for the metacestode in intermediate hosts was approved unanimously by the voters (median: 10).
**Germinal layer,** Expression	Inner cellular part of *Echinococcus* spp. metacestode, which includes several types of cells and produces several types of biological components of the metacestode (cyst fluid, brood capsules, and protoscoleces).	The term “layer” should be preferred to “germinal membrane” because of the complexity of the various “layers” of the cyst, and the possible confusion of “membrane” with the cell “membrane”, which has a specific definition in biology.	The correct description of the parasitic structures in the intermediate host includes three similar expressions, from inside to outside: Germinal layer Laminated layer Adventitial layer
			See also Figures 2A–2C.
* **Germinative cell**, Expression	Pluripotent somatic stem cell with some homologies but also differences to neoblasts of free-living flatworms. Germinative cells are the only mitotically active cells in the metacestode and give rise to all differentiated cells.	Most commonly used expression in the literature, with this definition [47].	The expression was approved by the majority of voters (median: 8) against the alternative “germinal” (median: 3).
		However, it must be noted that not all cells of the germinal layer are germinative cells.	
**Hooks,** Noun	Appendices of the adult form of *Echinococcus* spp. parasites that allow them to attach to the intestinal wall of the definitive hosts.	This term should be used because all hooks on the scolex of *Echinococcus* spp. have the same size.	
* **Hydatid,** Adjective	Refers to *Echinococcus* spp*.* metacestode in intermediate hosts; more specifically refers to the metacestode of *Echinococcus granulosus s.l.*	From the ancient Greek ὑδατίς –hydatis-, genitive – ὑδατίδος -hydatidos (vesicle/bladder full of water), “hydatid”, which describes the larval stage of *Echinococcus* spp. (metacestode) should never be used for the adult stage of *Echinococcus* spp.	The restriction of the definition of the adjective “hydatid” to the metacestode of *E. granulosus s.l.* was approved unanimously by the voters (median: 10).
		In order to avoid confusion between human diseases caused by the various species, “hydatid” should not be used to designate anything relating to alveolar echinococcosis (or *E. multilocularis*) or neotropical echinococcoses (or *E. vogeli* or *E. oligarthra*).	
**Hydatid,** Noun	Parasitological description of the cyst-like asexual larval form of cestodes; more specifically, description of the last stage of the metacestode of *E. granulosus s.l.*	From the ancient Greek ὑδατίς –hydatis-, genitive – ὑδατίδος -hydatidos (vesicle/bladder full of water). Usable with this definition only for purely parasitological descriptions.	Should be restricted (by usage, not strictly by definition) to the metacestode of *E. granulosus s.l*. It should not be used for the metacestode of *E. multilocularis.*
* **Hydatid fluid**, Expression	Liquid secreted by the germinal layer of *Echinococcus* spp. metacestode.	Following the suggestion of using “hydatid” only to qualify nouns or expressions related to *E. granulosus s.l.* (see above), this expression should also be restricted to these species. For any other species “fluid” should be preceded by the taxonomic name of the species, or the expression “cyst fluid” (for *in vivo* situations) or “vesicle fluid” (for *in vitro* situations) could be used.	All voters but one (median: 10) approved the use of “hydatid fluid” for *E. granulosus s.l.* only.
** **Immature (strobilar stage/adult form),** Adjective	Strobilar stage/adult form of *Echinococcus* spp. in the definitive host the last segment of which does not contain eggs (i.e. at least temporarily non-infectious to intermediate hosts).	“Immature” just indicates that the adult form has not fully developed to the stage of egg production; it does not imply that the adult worm will never become fertile, as could suggest the alternative “non-fertile”.	See also the entry “non-fertile” in Table 2B regarding the strobilar stage/adult form; and also “non-fertile”, approved for the metacestode in the intermediate host, in this table.
			This adjective was added by the SWG, on experts by suggestion, to the list of approved terms after the poll.
* **Infiltrate (periparasitic),** Noun	Histological components (cellular and fibrous) of host origin in alveolar echinococcosis; opposite to the “adventitial layer” of CE cysts, the cellular and fibrous infiltrate in AE has no clear limits with the surrounding liver parenchyma.	Alternative term to “infiltration”.	The use of “infiltrate” was approved by all voters but one (median: 10).
**Laminated layer,** Expression	Peripheral acellular outer part of *Echinococcus* spp. metacestode, mostly composed of mucopolysaccharides.	The laminated layer is produced by the parasite and exerts important functions in the interaction between the metacestode and the intermediate host [21].	The correct description of the parasitic structures in the intermediate host includes 3 similar expressions, from inside to outside: Germinal layer Laminated layer Adventitial layer
		The term “layer” should be preferred because of the complexity of the various “layers” of the metacestode, and the possible confusion of “membrane” with the cell “membrane”, which has a specific definition in biology.	See also Figures 2A–2C.
* **Larva,** Noun	Asexual reproduction stage of *Echinococcus* spp. parasites in their intermediate host.	Generally used as a popular equivalent of “metacestode”.	All voters but 2 considered that “larva” and “metacestode” were synonymous; however, metacestode should be preferred for scientific communication; see also the entry “larval form”.
**Larval form,** Expression	Asexual reproduction form of *Echinococcus* spp. parasites in their intermediate hosts.	Expression coherent with the recommended expression “adult form”.	The expression may be used as an equivalent of metacestode (or of larva) for communication to the public and to professionals.
** **Metacestode** (*singular*) **metacestodes** (*plural*)**,** Noun	Asexual reproduction form of *Echinococcus* spp. parasites in their intermediate hosts; second phase of development which includes all stages from the post-oncospheral stage to the fertile stage (with production of protoscoleces, if any).	Scientific designation of cestode parasites in their intermediate hosts; larva is an alternative noun usable for communication to the public and to professionals.	As the voters did not agree on the definition of “metacestode” (i.e. stages of development included in the definition), this was further discussed by experts after the poll, and the final definition (shown in the second column) was approved.
		The approved definition is supported by textbooks of veterinary parasitology [28] and those on *Echinococcus* spp. [72].	
* **Microcysts (echinococcal, *E. multilocularis*, AE),** Noun, **microcystic**, Adjective	Multiple small cysts (under 1 cm of diameter) with germinal layer, laminated layer and periparasitic infiltrate of host’s cells and fibrosis, characteristic of AE lesions (due to *E. multilocularis*).	Should be reserved for AE lesions *in vivo*; may be observed with selected imaging techniques such as Magnetic Resonance Imaging (T2-weighted images) in humans or preclinical models, and/or histological examination in experimental models.	In this situation, “micro” does not refer to “microscopic” but to “small” (from ancient Greek μικρός –micros (small)) macroscopic aggregated cysts in AE, opposite to the “big” –often isolated – cysts in CE or *E. oligarthra*-NE or multicystic presentation of *E. vogeli*-NE. This noun with this definition was approved by 8/10 voters (median: 9).
		Microcysts are parasitic structures, distinct from the central necrotic cavity often developed in the AE lesions (“pseudocyst” should be used for this cavity). See also the entry “pseudocyst” in this table.	
		The noun “microcysts”[6] may replace the expression “multiple small round cysts” found in Kodama et al., 2003[43].	
* **Non-fertile (metacestode, larva, cyst, microcyst…),** Adjective	Parasitic structure in the intermediate host that does not contain viable protoscoleces, and thus is unable to infect definitive hosts.	A “non-fertile” metacestode may be “viable”; these adjectives are not synonymous [22].	This definition of “non-fertile” for the metacestode in intermediate hosts was approved by all voters but one (median: 10)
* **Non-viable (metacestode, larva, cyst, microcyst, protoscolex…),** Adjective	Parasitic structure in the intermediate host that does not contain living cells able to proliferate in appropriate conditions, i.e. which does not grow when transplanted into a new intermediate host, or put into culture *in vitro*.	“Non-viable” implies that the parasitic structure does not grow when transplanted into the same or a new intermediate host, or put into culture in an appropriate medium *in vitro*. However, non-invasive evaluation of non-viability *in vivo* is still imperfect [22].	This definition of “viable” for the metacestode in intermediate hosts was approved by all voters (median: 10).
**Oncosphere,** Noun	Infectious form of *Echinococcus* spp. parasites resulting from the release from the egg through the action of proteolytic enzymes in the digestive system of intermediate hosts.	Should be restricted to the stage following ingestion by the intermediate host, just before the cell proliferation stage (post-oncospheral stage) that will constitute the metacestode.	See Figures 2A and 2B for detailed description and terminology [27].
**Pericyst,** Noun; **pericystic,** Adjective	Tissue surrounding the cyst. Regarding echinococcosis, the adjective applies to *E. granulosus sensu lato* only: tissue/structure (e.g. liver, lung, adrenal gland, brain, etc.) that surrounds the CE cyst which includes: the cyst fluid, the germinal layer, the laminated layer, the adventitial layer.	As the cyst, in *E. granulosus sensu lato* infection, includes the adventitial layer, the pericyst only corresponds to the organ parenchyma (plus vessels and ducts) which surrounds the cyst.	In the clinical (or animal/experimental) situation in CE, the noun “pericyst” (or the adjective “pericystic”) should not be used to designate the adventitial layer (which is “periparasitic”, not “pericystic”).
		Should not be used in *E. multilocularis* infection to qualify the tissue/structure that surrounds the lesions.	
**Periparasitic,** Adjective	Tissue surrounding the parasite. For *E. granulosus s.l.:* tissue/structures that surround the laminated layer of the hydatid.	In the cyst developed in *E. granulosus s.l.* infection in the intermediate host, the adventitial layer, produced by the host, surrounded by the normal parenchyma (plus vessels and ducts) represents the “periparasitic” tissue.	In the clinical (or animal/experimental) situation in CE, “periparasitic” (which includes the adventitial layer and the normal parenchyma of the organ) should not be a synonym for “pericystic” (which only includes the normal parenchyma of the organ; see also the definition of “(hydatid) cyst” and Figs. 2A–2C).
	For *E. multilocularis:* tissue/structures that surround the laminated layer of *E. multilocularis* microcysts.	In the lesion developed in *E. multilocularis* infection, the inflammatory tissue (granuloma) that surrounds the lesion without clear limit with the organ parenchyma represents the “periparasitic infiltrate”.	
* **Post-oncospheral stage**, Expression	Stage between the oncosphere and the fully constituted metacestode.	As it textually refers to the “oncosphere”, this expression more precisely addresses *Echinococcus* spp. than “transitional larva” which may apply to any type of larvae.	From a developmental biology/immunology point of view, the early stages of development in the intermediate host are crucial as this is the time when the parasite is most susceptible to killing [21, 76]; agreement on this point was unanimous (median: 10).
* **Proglottis** (*singular*), **proglottides** (*plural*)**,** Noun	Part of the adult form of *Echinococcus* spp. parasites resulting from the segmentation of the scolex in the intestine of definitive hosts.	Referring to the Greek origin of the word (γλῶττίς: strap), “proglottis (sing), proglottides (plur) should be preferred to “proglottid”; in British English, the original plural of words from other languages is kept, hence “proglottides”.	Both spellings “proglottis” and “proglottid” were in use. However, the majority of voters (median: 9) selected the original (Greek) spelling.
**Protoscolex (*plural:* protoscoleces),** Noun	Prefiguration of the scolex, produced by the brood capsules budding to the outside of the germinal layer of *Echinococcus* spp. metacestode and released in the cyst (CE) or micro-cyst (AE) fluid.	From the ancient Greek “σκὠλεξ”-scolex (worm), genitive: σκὠλεκος -scolecos, and not -scolicos; the plural form is σκὠλεκες, scoleces, and not scolices; with the prefix “πρῶτος”-protos (first/before).	For illustration of the formation of the protoscoleces, see Figure 1 in Koziol et al., 2016 [46].
**Scolex (*plural:* scoleces),** Noun	First segment (“head”) of the adult form of cestodes.	From the ancient Greek “σκὠλεξ”-scolex (worm), genitive: σκὠλεκος -scolecos, and not -scolicos; the plural form is ςσκὠλεκες, scoleces, and not scolices.	The plural form of scolex (and of all derived words) should definitely be “scoleces”.
**Segment,** Noun	Part of the adult form of *Echinococcus* spp. parasites resulting from segmentation of the scolex in the intestine of definitive hosts.	English equivalent of “proglottis”.	May be used as a popular equivalent of “proglottis” in reviews for students and professionals, or communication to the public, etc.
** **Strobilar stage,** Expression	Sexual reproduction stage (also called “adult form”) of *Echinococcus* spp. parasites in their definitive hosts.	Specific scientific expression to designate this step in the development of *Echinococcus* spp. cestodes.	Like its more popular synonym “adult form”, it includes all stages of development in the definitive host (fertile or immature worms i.e. with the last segment containing eggs or not).
**Suckers,** Noun	Appendices of the adult form of *Echinococcus* spp. parasites which allow them to attach to the intestinal wall of the definitive hosts and to feed from it.	No synonyms.	See also Figures 2A–2C.
* **Vesicle fluid**, Expression	Liquid produced *in vitro* by the metacestode of *Echinococcus* spp., regardless of the species.	Should be reserved for the *in vitro* situation where the fluid is devoid of host components, and when the *Echinococcus* spp.-derived nature is clear from the context.	The distinction between “vesicle fluid” (*in vitro* situation) and cyst fluid (*in vivo* situation) and usage of the noun “vesicle” for *in vitro* cultures only were approved by all voters but one (median: 10).
* **Vesicle (parasitic** or **echinococcal),** Noun	Anatomical entity produced by the growth *in vitro* of the metacestode of *Echinococcus* spp. regardless of the species.	Should be reserved for the *in vitro* situation where the vesicles are devoid of host components.	The distinction between “vesicle” (*in vitro*) and cyst (*in vivo*) and usage of the noun “vesicle” for *in vitro* cultures only, were approved by all voters but one (median: 10).
* **Viable (metacestode, germinal layer, larva, cyst, microcyst, protoscoleces…),** Adjective	Parasitic structure of the metacestode which contains living cells able to proliferate in appropriate conditions.	“Viable” implies that the structure (of any type) may grow in the same or a new intermediate host or in appropriate *in vitro* culture medium; viable parasitic structures may or may not contain protoscoleces (parasite stage necessary for the infection of definitive hosts). However, non-invasive evaluation of viability *in vivo* is still imperfect [22].	This definition of “viable” for the metacestode in intermediate hosts was approved by all voters (median: 10).
* **Worm,** Noun	Strobilar stage of *Echinococcus* spp. parasite in its definitive hosts (popular term).	Used as an even more popular equivalent of “strobilar stage” than “adult form”.	May be a source of confusion with non-parasitic worms.
			However, it is tolerated because it is useful for teaching, reviews for students and professionals, and communication to the public.

* Agreement obtained at the second stage of the consultation (the participants in the Consultation and Rating Group had to rate their approval or rejection of the words/expressions independently on a scale from 0 to 10; whenever relevant, the median of the votes and the nature of the agreement (majority or unanimity) are given in the “Comments” column

** Agreement to recommend a word that was not selected at the first stage of consultation; the approval was obtained at the second stage after further discussion between experts or after recommendation by the RRG.

**Table 1C:** Recommended terms for the clinical aspects of echinococcosis.

Word/expression	Definition	Reasons for acceptance, references, linguistic clarifications	Additional contributor’s opinion, if any
**Alveolar echinococcosis,** Expression (abbreviation: **AE**)	Disease due to *E. multilocularis,* with multiple microcysts giving an “alveolar” aspect on sectioning of the infected organ.	Widely used since the recognition of the disease in the 19th century [18, 78]. From the name of the species “*Echinococcus*” and the Latin adjective “alveolar”, genitive –alveolaris” (composed of or relating to “alveoli”/small holes/air sacs in the lung). The expression conforms to the recommendation of the WFP; the preceding adjective provides precision on the morphology of the lesion (alveolar). The adjective “alveolar” clearly indicates the morphological aspect of the lesions (especially in the liver).	Recommended by the WHO-IWGE since its first “Guidelines” [83] Differentiates the disease due to *E. multilocularis* from that due to *E. granulosus sensu lato* easily.
		Absence of precision regarding the type of disease leads to misunderstanding between experts, stakeholders and policy makers.	
* **Anti-parasitic treatment,** Expression	Pharmacological or biological treatment of echinococcosis, able to kill *Echinococcus* spp. or stop or delay their development at the various stages of the parasitic cycle.	May apply to the various stages of *Echinococcus* spp.-related infections, in the intermediate host (including humans) and in the definitive host (pets, domestic or wild animals). Type of drugs or treatment schedules may be different for the various stages and the various diseases [10, 35, 74, 75].	This expression was unanimously accepted by voters (median: 10) to replace definitively the noun “chemotherapy”, excluding all other options. See also Table 2C.
* **CE cyst,** Expression	Anatomical lesion due to infection with *Echinococcus granulosus sensu lato,* excluding all other species of *Echinococcus.*	Alternative name to designate a cyst/cysts due to infection with *E. granulosus sensu lato*. From the abbreviation of “cystic echinococcosis” (CE) and the ancient Greek noun κύστις, -cystis- (anatomical bladder). The expression should be used for clinical cases (cysts observed in cystic echinococcosis in humans or intermediate animal hosts) or *in vivo* experiments in animals [35, 81]. In agreement with the international WHO-IWGE classification of cysts due to *E. granulosus sensu lato* infection, from ultrasound examination (CE1 to CE5) [82].	A majority of voters (and a few RRG members) preferred to use “CE cyst” only and reject the use of hydatid cyst (median 8); however, there was no unanimity (3 voters rating under 6). Because the “Biology and Immunology subgroup supported the use of “hydatid” restricted to *E. granulosus s.l.,* at least temporarily, because of the widely used expressions “hydatid cyst” and “hydatid fluid”, both expressions will be tolerated. Using “CE cyst” is highly recommended.
* **Complicated (echinococcosis, CE, AE, cyst…),** Adjective	In CE, AE or NE: spontaneous or provoked events (including after therapeutic interventions) that occur in the CE/NE cyst/AE lesion.	Clinical definition. Complications may be: Rupture (outside or in structures of the infected organ), Compression or invasion of internal structures of the infected organ or of neighboring organs, Superinfection of the CE/NT cyst or of the AE pseudo-cyst with bacteria or fungi, Anaphylactic (IgE-dependent allergic) reactions due to echinococcal antigens, Compression or invasion of internal structures/tissues (e.g. for bone or brain) in CE/NE and AE [35].	The adjective “complicated”, for echinococcosis, should not be used to designate the simple growth of the parasite (with or without pain) if there are no consequences on the structures of the infected organ or on the neighboring organs. The large size of the cyst and its proximity to bile ducts or vessels in the liver (or bronchi or vessels in the lung, or vital structures in the brain) are not “per se” complications; even though they make the surgical treatment more difficult or impossible, they are just anatomical particularities.
			The term, with this definition, was accepted unanimously by the voters (median: 10).
**Cystectomy,** Noun	Removal of a cyst, whatever its nature; more specifically, removal of a CE cyst (anatomical lesion due to the infection by *E. granulosus s.l.* or *E. oligarthra*).	From the ancient Greek κύστις, -cystis- (anatomical bladder), and ἐκτομή, -ectomè/ectomia- (to cut out; to cut and remove), the suffix “-ectomy”, in surgical vocabulary, refers to any type of removal of an organ or a lesion. So, literally it means “removal of the cyst”. The context must be clear so that there may be no confusion between a CE cyst and any other types of cysts. In addition, the cystectomy should be qualified by the type of surgical technique, the type of cyst opening, and the extent of cyst removal.	Should never be used alone, without qualification, in a scientific/medical publication. The recommended order for qualification follows the AORC framework: Type of Approach (A): laparotomy, laparoscopy or robotic, Type of cyst Opening (O): opened-cyst (OC) or non-opened cyst (NOC), Type of Resection (R): cystectomy, hepatectomy (segmentectomy, lobectomy or pneumonectomy, for the lung). Completeness of cyst removal (C): total, subtotal or partial. See also Figure 3 for the description of the AORC framework with the definition of its various components in liver CE surgery.
**Cystic echinococcosis,** Expression (abbreviation: **CE**)	Disease due to *E. granulosus sensu lato.*	The expression conforms to the recommendation of the WFP; the preceding adjective, from the Middle French “*cystique*”, from the ancient Greek κύστις, -cystis- (anatomical bladder), provides precision on the morphology of lesion (cystic).	Recommended by the WHO-Informal Working Group on Echinococcosis (WHO-IWGE) since its first “Guidelines”[83]. Differentiates the disease due to *E. granulosus sensu lato*, from those due to *E. multilocularis*, *E. oligarthra* and *E. vogeli*, respectively.
		Absence of precision regarding the type of disease leads to misunderstanding between experts, stakeholders and policy makers.	
*/** **Cystoid,** Adjective (Adjective common to “Biology-Immunology” and to “Clinical Aspects”)	Hypodense, cyst-like image observed at CT in AE lesions.	From the ancient Greek eἶδος –eïdos- (shape), the suffix “-id” (with the infix “-o-” whenever useful in a given language) refers to anything “which has the shape of ...something”; thus “cystoid”: which has the shape of a cyst (without being a real cyst).	This adjective was proposed and published for the classification of AE lesions at computed tomography (CT) scanning in 2016 [23] and used thereafter in other publications. Although the adjective “pseudocystic” was preferred by the majority of voters (median: 9 for the “Biology-Immunology group and 7 for the “Clinical Aspects group), the adjective “cystoid” was maintained after the poll to describe cyst-like images observed using computed tomography scanning and which cannot be attributed unequivocally to the necrotic cavity present in advanced lesions of AE.
		In AE, the cystoid structures observed on imaging may be due to the central, liquefied necrosis in advanced AE lesions or to other cyst-like components such as conglomerate of microcysts or in very small lesions, occasionally, a hypodense solid necrosis.	
		As these structures do not correspond to a parasitological entity, a specific word should differentiate these pathological cyst-like macroscopic structures from the real parasitic “cysts” of CE.	
* **Daughter cyst,** Expression	Newly formed hydatids inside (and far less frequently outside, if any) the CE cyst in the development of *Echinococcus granulosus s.l.* Not applicable to *E. multilocularis*/AE.	This expression has been in use for a century, translated from the French “*vésicule fille*” (daughter vesicle), proposed by Dévé (the word “*vésicule*” being feminine in French, hence “daughter”). It is the established-by-use name of such anatomical structures which are derived from the germinal layer, in case of aggression of the metacestode (see Figs. 2A–2C) [18, 64, 68].	All suggested names for this metacestode structure were rejected by the majority of voters (median for “daughter cyst”, daughter hydatid, and daughter vesicle: 2, 1 and 3, respectively; means 4.63, 1.72 and 4.70, respectively).
			The word “daughter” evokes sexual reproduction (which does not exist for the metacestode), and “cyst” a complete CE cyst structure, with a fully developed adventitial layer (which is generally hardly present in this structure). However, the adjective “secondary” was considered by the participants in the CRG to be irrelevant for this situation (used for the growth of new cysts after protoscolex spillage), and the noun “vesicle” was accepted by biologists with restricted use for *in vitro* culture of the metacestode; hence the decision of the SWG to keep the “historical”, albeit inexact, wording.
* **Disseminated (CE or AE, or NE),** Adjective	Clinical form of cystic or alveolar or neo-tropical echinococcosis disseminated to more than one organ/tissue.	The definition implies that the cysts/lesions are not in a single organ; it does not assume the nature of dissemination (e.g. multiple location of infection; local/regional invasion; metastasis)	This definition was accepted by the majority of voters (median: 10)
**Echinococcosis** (*plural:* **Echinococcoses**), Noun	Disease(s) related to infection with parasites of the *Echinococcus* (*E.*) genus.	Recommended by the World Federation of Parasitologists (WFP) (i.e. name of the genus followed by the suffix -osis) The English name of the World Association of Echinococcosis (WAE; previously “International Association of Hydatidology) was modified in 2015 at the 26th World Congress of Echinococcosis in Bucharest, Romania, to follow this rule.	Without precision regarding the type of disease, this noun can only be used when all diseases due to *Echinococcus* spp. are considered together, and preferably used as a plural (echinococcoses); it should be avoided in epidemiological, clinical, and mechanistic studies covering all 3 diseases. Besides this “generic” usage, distinction should always be made between cystic echinococcosis (CE), due to *E. granulosus sensu lato (s.l.*), alveolar echinococcosis (AE), due to *E. multilocularis*, and neotropical echinococcosis (NE), due to *E. vogeli* or *E. oligarthra.*
**ERCP,** Acronym for **Endoscopic Retrograde Cholangio-Pancreatography,** Expression	Per-endoscopic technique used to explore the biliary and pancreatic ducts, regardless of the disease; it may or may not be associated with sphincterotomy or other additional procedures.	In CE and AE diagnostic procedures, no exploration of the pancreatic ducts is performed, but the acronym ERCP is commonly used even though the pancreatic exploration is not performed.	Performance of the ERCP may be only for diagnostic purposes; however, usually it has a therapeutic intent and is the first step of perendoscopic biliary drainage. In scientific publications, this distinction should be clearly stated.
***Ex-vivo* liver resection with auto-transplantation (ELRA),** Expression	Surgical operation which includes: Total hepatectomy followed by Ex-vivo resection of the pathological parts of the liver associated with Various types of reconstruction of the bile ducts and/or vessels then by Re-implantation (“auto-transplantation”) of the liver orthotopically.	Surgical technique used for the treatment of advanced AE. The expression includes all steps of the procedure; so it should be preferred to partial expressions such as “ex-vivo liver resection” or “auto-transplantation”. The abbreviation was proposed by the surgical team that has performed most of these procedures and includes all main terms of the expression [2].	The numerous alternative expressions and acronyms should no longer be used.
**Hepatectomy**	Removal of the liver or part of it.	From the ancient Greek ἧπαρ, -hepar; genitive ἥπατος –hepatos (liver), and the suffix ἐκτομή, -ectomè/ectomia- (to cut out; to cut and remove); in the surgical vocabulary it refers to any type of removal of an organ or a lesion. So, literally it means “removal of the liver” (which is literally correct only in liver transplantation). Various types are described, valid for all types of diseases subject to liver surgery.	Should always be qualified, according to the usual definitions of hepatic surgery, and of the liver segments; definitions are valid for the application to all types of diseases, including echinococcosis [29].
* **Hydatid cyst,** Expression (word common to “Biology – Immunology” and to “Clinical aspects”)	Anatomical lesion due to infection with *Echinococcus granulosus sensu lato,* excluding all other species of *Echinococcus.*	From the ancient Greek ὑδατίς –hydatis-, genitive – ὑδατίδος -hydatidos (vesicle/bladder full of water). According to the definition of “hydatid” (as an adjective), the expression should be restricted to designate a cyst/cysts due to infection with *E. granulosus s.l.* in the intermediate host (including humans).	As it adds to confusion in epidemiological and clinical studies, it should never be used to designate the anatomical lesion due to *E. multilocularis* infection. The definition was approved by the unanimity of voters (median: 10). A majority of voters preferred to totally reject the use of “hydatid cyst” (median 8 for the specific question; see above “CE cyst”). As there was no unanimity and as the “Biology and Immunology” subgroup supported the use of “hydatid” restricted to *E. granulosus s.l.,* at least temporarily, because of the widely used expression “hydatid cyst”, both expressions will be tolerated. Nevertheless, the use of “CE cyst” is highly recommended.
		As it designates an anatomical lesion and not a disease, it should never be used as an alternative name for *Echinococcus* spp.-related infection (“echinococcosis”). To designate the diseases due to *Echinococcus* spp. the noun “echinococcosis” qualified by the type of disease (cystic, alveolar or neotropical) should be used (abbreviations CE, AE, NE; see above).	
** **Laparoscopy,** Noun	Surgical approach performed for operations in the abdomen or pelvis using small incisions (usually 0.5–1.5 cm) with the aid of a camera.	Qualifies any surgical intervention performed under laparoscopy. To be preferred to “coelioscopy”, more used in gynecology.	Should be added to the noun or expression describing any type of intervention, if this surgical intervention is performed by laparoscopy. See also Figure 3 and the description of the AORC framework with the definition of its various components in liver CE surgery.
** **Laparotomy,** Noun	Surgical approach which includes a large opening of the abdomen.	Qualifies any surgical intervention performed under laparotomy.	Should be added to the noun or expression describing any type of intervention, if this surgical intervention is performed by laparotomy. See also Figure 3 and the description of the AORC framework with the definition of its various components in liver CE surgery.
**Modified catheterization technique,** Expression **Mo-CAT,** Acronym	Percutaneous procedure for treatment of CE cysts that results in removal of the cyst layers, possibly including daughter cysts, in addition to the cyst fluid and protoscoleces.	Similar techniques (such as PEVAC) previously described are no longer in use [36, 71, 81]. The name of the procedure and the acronym “Mo-CAT” clearly distinguish this procedure from the conventional PAIR and from the “standard catheterization technique” [3, 61]	The mode of percutaneous puncture guidance (e.g. ultrasound-guided, CT-guided) should also be given, as well as the type/size of catheterization and aspiration.
			The expression and its acronym were accepted by the majority of voters (median: 10).
* **Multicystic (echinococcosis; images),** Adjective	Any type of echinococcosis with multiple aggregated cysts observed on imaging or during surgery in the same organ.	Avoids the confusing use of “polycystic”, which currently refers both to cystic or alveolar echinococcosis with multiple large size-cysts observed on imaging, and to *E. vogeli* infection; also avoids confusion with polycystic liver (and kidney) disease.	The adjective “multicystic” should not be used to qualify the usual “alveolar” aspect of the aggregated “microcysts” in AE. It should not be used to qualify cases of CE or NE with multiple separated and independent cysts observed on imaging or during surgery in the same organ or different organs; see the definition of “multiple cyst-”. This term was accepted by the majority of voters (median: 10). See also “polycystic”, the rejected term, in Table 2C.
** **Multiple cyst-,** Expression used as Adjective	Any type of echinococcosis (except AE) with multiple separated and independent cysts observed on imaging or during surgery in the same organ or different organs.	Clearly distinguishes the “multicystic” aspect of lesions and the presence of multiple cysts.	Should not be used to qualify cases of AE with multiple lesions. Should not be used to qualify cases with aggregated cysts in the same organ (i.e. “multicystic” CE or NE); see the definition of “multicystic”.
**Neotropical echinococcoses,** Expression (abbreviation: **NE**)	Diseases due to *E. vogeli* or *E. oligarthra.*	Conforms to the recommendation by the WFP (echinococcosis); the preceding adjective provides precision on the particularity of the diseases (neotropical) [36]. “Neotropical” evokes the unique geographic distribution of the *Echinococcus* spp. involved (in the tropical areas of the New World).	Although the expression does not give any information on the morphology of the lesion (“polycystic” in *E. vogeli* infection; “unicystic” in *E. oligarthra* infection), it has been in use for a while in the literature and was approved by the South American Working Group on *E. vogeli* and *E. oligarthra*; it usefully distinguishes the diseases caused by these species from CE and AE (see definitions above).
** **New CE cyst(s),** Expression	Appearance of a new CE cyst in a location different from where a CE cyst was diagnosed before.	The definition covers all new cysts that have appeared in a patient after a first diagnosis; the new cyst may have appeared spontaneously (e.g. during a “Watch and Wait” period of follow-up) or after any type of treatment.	This expression was added by the SWG after the poll, in agreement with the writers of the WHO-IWGE Technical Manual. It excludes all cases of cyst recurrence or reactivation, as well as “secondary echinococcosis” (see the definitions of “reactivation” “recurrence”, and secondary echinococcosis” in this Table 1C).
** **Non-opened cyst (total cystectomy),** Expression	Surgical operation which does not include cyst opening before cyst removal (cystectomy). Only applies to CE, and for total cystectomy; it is not relevant for AE.	Linguistically more correct than “close(d) cystectomy” (a cyst can be “non-opened” during the surgical procedure, a cystectomy cannot be close or closed) [81]. This expression clearly describes the situation of the cyst (non-opened) and the highly reduced risk of dissemination of protoscoleces or germinal membrane fragments related to the surgical procedure.	Newly introduced expression, resulting from the discussion between members of the “Clinical aspects” subgroup of the CRG.
			The adjective expression “non-opened cyst”, its acronym “NOC” and its definition, to qualify more precisely the total cystectomy, were accepted by a majority of voters (median: 10) See also Figure 3 and the description of the AORC framework with the definition of its various components in liver CE surgery.
** **Opened cyst (total, subtotal, partial cystectomy),** Expression **OC**, Acronym	Surgical operation which includes cyst opening before cyst removal (cystectomy). Only applies to CE; may be at first or second intent; it is not relevant for AE.	Linguistically more correct than “open cystectomy” (a cyst can be opened during the surgical procedure, a cystectomy cannot) [81].	Newly introduced expression, resulting from the discussion between members of the “Clinical aspects” subgroup of the CRG. The adjective expression “opened cyst”, its acronym “OC”, and its definition, to qualify more precisely the total cystectomy, were accepted by a majority of voters (median: 10) See also Figure 3 and the description of the AORC framework with the definition of its various components in liver CE surgery.
		This expression clearly describes the situation of the cyst (opened) and the potential risk of dissemination of protoscoleces or germinal membrane fragments related to the surgical procedure.	
**(Orthotopic) Liver transplantation,** Expression OLT, Acronym	Removal of the recipient’s liver followed by the transplantation of the donor’s liver (or part of liver) at the same anatomical location.	Surgical technique used for the treatment of advanced cases of AE, and more rarely of CE. Often used for “allo-transplantation of the liver”; does not include auto-transplantation.	The adjective “orthotopic” (and its abbreviation “O”) is facultative, since the great majority of liver transplantations in humans are orthotopic.
**PAIR**, Acronym for **Puncture, Aspiration, Injection of protoscolecide, Reaspiration,** Expression	Percutaneous treatment of CE cysts using needle puncture and use of protoscolecide agents.	Accepted name and acronym to designate the procedure, since its description by Ben Amor et al., 1986 [8] in French (same acronym), and its first publication in English by Gargouri et al., in 1990 [20]. It describes the 4 steps of the procedure which does not include catheterization of the cyst. The procedure was evaluated and its indications clarified by the WHO-IWGE [10].	The name and acronym must be used only for the initially described procedure, with needle puncture, manual syringe aspiration, without catheterization or other associated techniques.
* **Partial (cystectomy),** Adjective	Surgical intervention that only partially removes the 3 layers of the CE cyst.	Opposite to “subtotal” which only leaves parts of the adventitial layer in place, this may include a partial removal of any layer (including parts of the germinal layer and laminated layer); usually only the adventitial layer is left.	By definition, in this situation, the CE cyst has to be opened (first or second intent opening). The adjective and its definition were accepted by a majority of voters (median: 8); however, a complementary discussion was necessary to precisely agree on the differences between “subtotal” and “partial”. See also Figure 3 and the description of the AORC framework with the definition of its various components in liver CE surgery.
*/** **Percutaneous pseudocyst drainage,** Expression	Imaging-guided percutaneous transhepatic interventional technique used for the drainage of the central “pseudocyst” in advanced lesions of AE. Applies to AE only.	For the treatment of AE, this expression is preferred to “cavity drainage” since it refers to the typical “pseudocyst” resulting from the necrosis of AE lesions; “cavity drainage” is more commonly used for the drainage/treatment of the postoperative cavities after surgery for CE (cystectomy). In AE, such drainage has no temporal relationship with any other interventional treatment.	The expression was accepted by the majority of voters (median: 10). No acronym was fixed. In any description of the procedure, the mode of percutaneous puncture guidance (e.g. ultrasound-guided, CT-guided) should also be given, as well as the type/size of catheterization and aspiration.
** **Percutaneous post-surgery cavity drainage,** Expression	Imaging-guided percutaneous transhepatic interventional technique used for the drainage of post-operative cavities after surgery for CE. Applies to CE only.	A clearly different expression for the drainage of the cystoid necrotic cavity in AE *versus* the treatment of CE with postoperative residual cavity was considered useful. The “post-surgery” temporal performance of such drainage is clearly indicated in the expression.	This expression was added by the SWG after the poll, after recommendation by the RRG, in order to clearly distinguish the respective situations in AE and CE. In any description of the procedure, the mode of percutaneous puncture guidance (e.g. ultrasound-guided, CT-guided) should also be given, as well as the type/size of catheterization and aspiration.
**Perendoscopic biliary drainage** Expression	Non-surgical interventional technique used for the drainage of the biliary tree, through ERCP.	The procedure, which applies both to CE and AE, may or may not include biliary stenting [4, 71].	The procedure does not include a curative action on the CE cyst or on the AE lesion; it only treats complications of the diseases and/or of their surgical treatment. In scientific publications, if biliary stenting is associated with the procedure, this should be specifically mentioned, as well as the type, size, and number of stents.
* **Peri-adventitial (cystectomy),** Adjective	Total cystectomy performed without opening the cyst, and which uses the dissection space between the adventitial layer and the “normal” liver parenchyma to remove the cyst totally.	The adjective indicates more precisely that the resection is performed outside the adventitial layer (i.e. the adventitial layer is included in the resected cyst) [37, 81].	The use of this adjective for the description of cystectomy is facultative. The adjective “peri-adventitial” and its definition, to qualify more precisely the total cystectomy, were accepted by a majority of voters (median: 10) See also Figure 3 and the description of the AORC framework with the definition of its various components in liver CE surgery.
**Protoscolecide*,*** Noun **protoscolecidal,** Adjective	Compound (natural or chemical) which is able to kill the protoscoleces.	From the ancient Greek “σκὠλεξ”-scolex (worm), genitive: σκὠλεκος -scolecos, and not -scolicos; plural σκὠλεκες, scoleces, and not scolices), with the prefix “πρῶτος”-protos (first/before), and the Latin suffix – “-cide”, from “caedere” (to kill).	Should be used instead of “scolicide”, “scolecide” and “protoscolicide”. See also Table 2C, about the rejected alternative words.
*/** **Pseudocyst,** Noun, and **pseudocystic,** Adjective (Noun and Adjective common to “Biology-Immunology” and to “Clinical Aspects”)	Irregular cyst-like anatomical entity due to the central necrosis in AE lesions at advanced stages.	From the ancient Greek “ψευδής” –“pseudès- (= false, deceptive, misleading) that designates something which looks like something but is not; thus “pseudocyst”: anatomical entity which looks like a cyst but is not a cyst. The terms differentiate the cyst-like necrotic cavity in AE from the real “cyst” of CE (with its parasitic structure including the 3 layers). This structure does not correspond to a parasitological entity. In medicine, the term “pseudocyst” is generally used for the necrotic cavities that develop after episodes of pancreatitis in the pancreas.	The terms were approved by the majority of voters (median: 9). The context and the organ being different from pancreatitis, and the formation of the cavity also being due to liquefied necrosis of an inflammatory lesion, it was considered that the words “pseudocyst” and “pseudocystic” could also be used in AE. However, the adjective “cystoid” was maintained to qualify cyst-like structures (irrespective of their anatomical nature) observed at imaging (especially at CT-scan) in AE lesions.
**PTBD,** Acronym, **for Percutaneous Transhepatic Biliary Drainage,** Expression	Non-surgical interventional technique used for the drainage of the biliary tree, which is used in AE or complicated CE, after percutaneous puncture.	No synonyms. Expression and acronym widely used in the medical literature, whatever the application. The procedure applies both to CE and AE and does not include a curative action on the CE cyst or on the AE lesion.	The mode of percutaneous puncture guidance (e.g. ultrasound-guided, CT-guided) should also be given, as well as the type/size of catheterization.
** **Reactivation,** Noun	Appearance of daughter cyst(s) in the solid matrix of spontaneously inactivated CE4 cysts that shows an evolution towards a CE3b stage.	The term “reactivation” is reserved for spontaneously inactivated cysts (observed after an initial diagnosis of CE4 cyst, with or without formal “Watch and Wait” follow-up).	This term was added by the SWG after the poll, in agreement with the writers of the WHO-IWGE Technical Manual, in order to clearly distinguish the various situations regarding new lesions or changes in lesions that appeared spontaneously or after attempts at treatment.
** **Recurrence,** Noun	Appearance of an active CE cyst (types CE1-3, usually CE3b) in the same location where a treated cyst was located, independently of the type of previous treatment.	Following its definition in oncology, the term “recurrence” is reserved for the post-therapeutic situation; the term “reactivation” applies to non-treated cysts.	This term was added by the SWG after the poll, in agreement with the writers of the WHO-IWGE Technical Manual, in order to clearly distinguish the various situations regarding new lesions or changes in lesions that appeared spontaneously or after attempts at treatment.
			NB: the occurrence of a post-surgical cavity needs to be excluded before a diagnosis of recurrence is made (cf. the definition of “percutaneous post-surgical cavity drainage”, in this Table 1C).
** **Secondary cyst,** Expression	Newly formed cysts from the dissemination of protoscoleces or germinal layer fragments, in the serosal cavities, spontaneously, accidentally or after surgery or cyst puncture.	Mode of formation distinct from that of daughter cysts; the adjective “secondary” should, however, be restricted to the appearance of new CE cysts in the peritoneum, pleura or meningeal space because of spontaneous or per-treatment spillage of the cyst content.	This expression was added by the SWG after the poll, to clearly distinguish this situation from the development of “daughter cyst”. The expression was approved by the writers of the WHO-IWGE Technical Manual, in order to clearly distinguish the various situations regarding new CE cysts or changes in CE cysts that appeared spontaneously or after attempts at treatment.
* **Standard catheterization (technique),** Expression **S-CAT,** Acronym	Modification of the PAIR technique which includes the insertion of a catheter left or not in the cyst temporarily for the treatment of selected CE cysts.	The name of the procedure and the acronym “S-CAT” clearly distinguish this procedure from the conventional PAIR and from the “modified catheterization technique” (“Mo-CAT”) [3, 35].	The expression and its acronym were accepted unanimously by the voters (median: 10) It was also proposed to complete the description by the following mentions: One-session S-CAT: the catheter is removed during the first procedure; Multiple-session S-CAT: the catheter is removed at another session.
			The mode of percutaneous puncture guidance (e.g. ultrasound-guided, CT-guided) should also be given, as well as the type/size of catheterization.
*/** **Sub-total (cystectomy),** Adjective	Nearly total cystectomy with incomplete removal of the adventitial layer of a CE cyst	Situation that occurs when limited parts of the cyst cannot be removed safely because of the proximity of blood vessels or other anatomical structures (e.g. bile ducts, bronchi, brain anatomical structures with critical functions). To be qualified of “subtotal”, the cystectomy must remove the totality of the germinal and laminated layer; only limited parts of the adventitial layer are left.	By definition, in this situation (as in “partial cystectomy), the cyst has to be opened (second intent opening). The adjective and its definition were accepted by a majority of voters (median: 10); however, a complementary discussion after the poll was necessary to precisely agree on the differences between “subtotal” and “partial”.
			See also Figure 3 and the description of the AORC framework with the definition of its various components in liver CE surgery.
* **Total (cystectomy),** Adjective	Complete removal of a CE cyst, including the content (fluid and protoscoleces) and all layers of the cyst (germinal, laminated and adventitial layers.	The adjective “total” insists on the complete removal of the CE cyst by the surgical operation (the “C”, for “completeness” of the AORC framework). It does not prejudge of the opening or not of the cyst at any time of the procedure. This operation, when performed on a non-opened cyst, was sometimes called “peri-cystectomy”; however, a proper definition of the CE cyst is against the use of this word (see Tables 1B and 2C and Figs. 2A–2C).	The adjective “total”, speaking of cystectomy, and its definition were accepted by all voters but one (median: 10). See also Figure 3 and the description of the AORC framework with the definition of its various components in liver CE surgery.

* Agreement obtained at the second stage of the consultation (the participants in the Consultation and Rating Group had to rate their approval or rejection of the words/expressions independently on a scale from 0 to 10; whenever relevant, the median of the votes and the nature of the agreement (majority or unanimity) are given in the “Comments” column.

** Agreement to recommend a word that was not selected at the first stage of consultation; the approval was obtained at the second stage after further discussion between experts or after recommendation by the RRG.

*/** Agreement obtained at the second stage of the consultation; however the final definition was obtained after further discussions with the members of the RRG.

**Table 2A:** Rejected terms for the genetics and epidemiology of *Echinococcus* species.

Word/expression	Definition	Reasons for rejection, references, linguistic clarifications	Comments
***Alveococcus,*** Noun	Non-valid genus name often used in the Russian/ex-Soviet Union literature to include *Echinococcus multilocularis* (Leuckart, 1863) which actually belongs to the *Echinococcus* genus.	*E. multilocularis* (Leuckart, 1863) belongs to the *Echinococcus* genus [50, 67]. The International Code for Zoological Nomenclature should be followed (see Table 1A) [52].	The genus *Alveococcus* Abuladze, 1959 (including *Alveococcus multilocularis* (Leuckart, 1863)), still used occasionally, particularly in Russian literature, was erected to separate *E. multilocularis* from the other species. There is no longer a taxonomic basis for this different genus name.
		Only those approved species names in the genus *Echinococcus* mentioned in Table 1A should be used.	
***Echinococcus alveolaris,*** Expression	Non-valid species name sometimes – and even recently – used in the German and Turkish literature to designate either the species *Echinococcus multilocularis* or the disease “alveolar echinococcosis”[60].	To designate the cestode, the International Code for Zoological Nomenclature should be followed (see Table 1A)	Should definitively be abandoned.
		To designate the disease, the recommendations of the World Federation of Parasitologists (WFP) should be followed (see Table 1C)	
***Echinococcus cysticus,*** Expression	Non-valid species name sometimes used in the German and Turkish literature to designate either the species *Echinococcus granulosus s.l.* or the disease “cystic echinococcosis”[44]	To designate the cestode, the International Code for Zoological Nomenclature should be followed (see Table 1A)	Should definitively be abandoned.
		To designate the disease, the recommendations of the World Federation of Parasitologists (WFP) should be followed (see Table 1C).	
***Echinococcus granulosis***	Non-valid species name with wrong orthography, often found in publications.	To designate the cestode, the International Code for Zoological Nomenclature should be followed (see Table 1A).	Simply wrong.
***Echinococcus granulosus sensu lato* species (non-exhaustive, following alphabetic order),** Synonymous expressions: ***E. borealis*** ***E. cameroni*** ***E. cepanzoi*** ***E. intermedius*** ***E. longimanubrius*** ***E. lycaontis*** ***E. minimus*** ***E. patagonicus***	Non-valid historical or recently proposed species names for *Echinococcus* spp. within the *Echinococcus granulosus sensu lato* complex that were later synonymized with the currently recognized species.	Recently proposed species names should not be used, unless a case can be made (e.g. by molecular studies) that they merit recognition as separate species.	Only those approved species names in the genus *Echinococcus* mentioned in Table 1A should be used.
			Pending precise definition of additional species within *Echinococcus granulosus sensu lato*, besides the currently accepted “species” (see Table 1A), “G” genotypes should be used [52–54, 57, 66].
***Echinococcus oligarthrus, Echinococcus oligarthus,*** Expressions	Non-valid names of the species among *Echinococcus* spp. which is responsible for one of the clinical forms of “neotropical echinococcoses”	Often referred to as *E. oligarthrus* (or even *oligarthus)*, the species was originally described as *Taenia oligarthra* (Diesing, 1863) by Diesing [14].	In this case, there was no mistake in the original name of species given by Diesing; variants were used mistakenly afterwards in the scientific literature. However, the genus “*Echinococcus*” was subsequently identified and replaced the genus “*Taenia*”.
		Linguistic and historical arguments are convincing [52, 58, 81]. See Table 1A.	
		The component ἄρθρα – *arthra* (joints) – is the plural of ἄρθρον -*arthron* (joint). The name is therefore not an adjective, but a noun in apposition, which does not change its ending according to the gender of the generic name. This was recognized earlier but subsequently ignored.	
***Echinococcus sibiricensis* (Rausch & Schiller, 1954)*,*** Expression	Non-valid historical name for *Echinococcus multilocularis.*	*E. multilocularis* (Leuckart, 1863) is currently the only accepted name in the international nomenclature [18, 50, 63, 77].	The species described as *E. sibiricensis* (Rausch & Schiller, 1954), was subsequently found to be conspecific with *E. multilocularis* (Leuckart, 1863) [63].

**Table 2B:** Rejected terms for the biology and immunology of *Echinococcus* species.

Word/expression	Definition	Reasons for rejection, references, linguistic clarifications	Comments
* **Adult worm**, Expression	Sexual reproduction stage *Echinococcus* spp. parasites in their definitive hosts.	Sometimes used as equivalent of “adult form”.	The expression was rejected by a majority of voters (median: 3).
		Adult worm may be considered redundant, since worms are adult forms of helminths.	
		In addition, “adult form” in the definitive host corresponds to “larval form” in the intermediate host.	
* **Adventitia,** Noun	Fibrous and cellular layer between the laminated layer of the hydatid in *E. granulosus s.l.* infection and the normal parenchyma of the host organ where the metacestode developed.	Word of Latin origin, “adventitia” is the outermost connective tissue covering of an organ, vessel, or other biological structure. In the CE cyst, this poorly cellular and mostly fibrous layer results from the host immune reaction to *E. granulosus sensu lato* metacestode.	As “adventitia” may be used for any biological structure, the use of “adventitial layer”, which refers to the parasite “germinal layer” and “laminated layer”, was preferred by the voters for this structure of host origin (median: 2, for “adventitia”).
* **Boundary (periparasitic),** Noun	Structures that surround the laminated layer of the hydatid of *Echinococcus* spp. metacestodes in their intermediate hosts, at the border with the organ parenchyma.	Alternative noun to “border”; “border” is more common in pathology terminology.	Rejected because of usage (median: 0).
* **Died-out (cyst, lesion),** Adjective	Non-viable parasitic structure as evidenced by imaging (complete calcification in AE, CE5 cyst in CE) or histological examination (absence of viable parasitic cells).	Could be used in clinical or experimental situations when evidence of absence of viability is not obtained by transplantation or *in vitro* culture.	Rejected by a majority of voters (median: 10, for rejection).
* **Germinal cell**, Expression	Pluripotent somatic stem cell with some homologies but also differences to neoblasts of free-living flatworms. Germinative cells are the only mitotically active cells in the metacestode and give rise to all differentiated cells.	Alternative expression to designate germinative cells. Same adjective as in the expression “germinal layer” where these cells are located. However, not all cells in the germinal layer correspond to this definition.	The germinal layer also contains other cell types such as muscle cells, nerve cells, and calcareous corpuscle-producing cells. This was an argument to reject the adjective “germinal” for such cells (median: 3).
**Germinal membrane,** Expression	Inner cellular layer of the *Echinococcus* spp. metacestode.	This term should be avoided and the term “layer” should be preferred because of the complexity of the various “layers” of the hydatid, and the possible confusion of “membrane” with the cell “membrane”, which has a specific definition in biology.	Germinal layer is the recommended expressions to designate the inner cellular “layer” of parasite origin in the *Echinococcus* spp. metacestode.
**Hooklets,** Noun	Appendices of the adult worm of *Echinococcus* spp. parasites allowing them to attach to the intestinal wall of the definitive hosts.	This term, a diminutive of “hooks”, is also used to designate these structures of the scolex and adult form of *Echinococcus* spp.; it should be avoided because the term “hooklet” infers a smaller version of a hook, i.e. that two forms are present, a larger “hook” and a smaller “hooklet” as in the haptor of some Monogenean parasites.	“Hook” is the only term recommended.
**Hydatic,** Adjective	Related to *Echinococcus* spp.	Gallicism for “hydatid” used as an adjective.	Should not be used in English.
* **Infiltration (periparasitic),** Noun	Histological components (cellular and fibrous) from host origin in alveolar echinococcosis	Alternative noun to “infiltrate”; infiltrate is more common in pathology terminology; infiltration has a slightly different meaning (systemic homing of inflammatory in the whole organ).	Rejected because of usage (median: 0).
**Laminated membrane,** Expression	Outer acellular layer of the *Echinococcus* spp. metacestode.	This term should be avoided and the term “layer” should be preferred because of the complexity of the various “layers” of the hydatid, and the possible confusion of “membrane” with the cell “membrane”, which has a specific definition in biology.	Laminated layer is the recommended expression to designate the outer acellular “layer” of parasite origin in the *Echinococcus* spp. metacestode.
**Neoblast**, Noun	Somatic stem cell type which shares homologies with germinative cells from *Echinococcus* spp. metacestodes, but also display significant differences.	Specific of planarians (and other free-living flatworms).	Should not be used for *Echinococcus* spp.
** **Non-fertile (adult form or worm),** Expression	Adult form/worm of *Echinococcus* spp. whose last segment does not contain eggs.	Alternative to “immature” (adult form or worm). However, non-fertile could imply that this worm is not able to become fertile (which is wrong for the majority of adult *Echinococcus* spp. forms, especially in the definitive host *in vivo*).	*In vitro*-reared adult forms of *Echinococcus* spp. do not contain eggs; they exhibit some differences from the *in vivo* developed adult forms, and they are presumably non-fertile [56].
			However, the adjective “non-fertile” cannot be accepted for the usual situation of *in vivo* developed *Echinococcus* spp. adult forms/worms.
**Primary cells**, Expression	All cells that result from lytic digestion of the *Echinococcus* spp. metacestode and can be kept in culture using the “primary cell cultivation system”.	“Primary cell” is not a specific cell type of *Echinococcus* spp. metacestode, but a “culture system” [70].	Primary cells are a mixture of “germinative cells” (~80% at the beginning), muscle cells and nerve cells [45].
* **Proglottid,** Noun (*singular*), **Proglottids** (*plural*), Noun	Orthographic variant of “proglottis”: part of the adult form of *Echinococcus* spp. parasites resulting from segmentation of the scolex in the intestine of definitive hosts.	Proglottid is an alternative to proglottis in several dictionaries; the plural “proglottids” is commonly used in the USA (American English). Referring to the Greek origin of the word, “proglottis (sing), proglottides (plur) should be preferred.	This spelling was rejected by a majority of voters (7/10; median: 3)
**Protoscolices** (Plural of protoscolex), Noun	Prefiguration (“proto”) of the “scolex”, produced by the brood capsules budding from the germinal layer of *Echinococcus* spp. metacestode and released in the cyst fluid.	From the ancient Greek “σκὠλεξ”-scolex (worm), genitive: σκὠλεκος -scolecos, and not -scolicos; the plural form is σκὠλεκες, scoleces, and not scolices; with the prefix “πρῶτος”-protos (first/before).	Protoscoleces is the correct plural form, according to the etymology of the word.
**Scolices** (Plural of scolex), Noun	First segment (“head”) of the adult form of cestodes.	From the ancient Greek “σκὠλεξ”-scolex (worm), genitive: σκὠλεκος -scolecos, and not -scolicos; the plural form is σκὠλεκες, scoleces, and not scoleces.	Scoleces is the correct plural form, according to the etymology of the word.
**Totipotent somatic stem cell,** Expression	Stem cells in the germinal layer of *Echinococcus* spp. metacestode.	Alternative to “germinative cells”; however, the real totipotent cell, with specific markers, has not been identified yet [45].	Pending a better definition of the really totipotent cells in the germinal layer of *Echinococcus* spp. metacestode, a single expression “germinative cell” should be kept.
* **Transitional larva**, Expression	Stage of *Echinococcus* spp. between the oncosphere and the fully developed hydatid (i.e. first stage in the metacestode development).	Alternative expression to “post-oncospheral” stage, but less precise to qualify a stage of a metacestode since it may apply to any larva. In addition, “transitional” may qualify any other stages of development (e.g. when brood capsules produce protoscoleces).	Rejected by all voters but 2 (median: 0).

* Agreement to reject the term obtained at the second stage of the consultation (the participants in the Consultation and Rating Group had to rate their approval or rejection of the words/expressions independently on a scale from 0 to 10; whenever relevant, the median of the votes is given in the “Comments” column).

** Agreement to reject a term that was not selected at the first stage of consultation; the agreement was obtained at the second stage after further discussion between experts or after recommendation by the RRG.

**Table 2C:** Rejected terms for the clinical aspects of echinococcosis.

Word/expression	Definition	Reasons for rejection, references, linguistic clarifications	Comments
* **Adventitial (cystectomy),** Adjective	Total cystectomy (usually performed without opening the cyst) which uses the dissection space between the adventitial layer and the “normal” liver parenchyma to remove the cyst more easily.	Proposed alternatives to “peri-adventitial” to qualify this surgical technique [35].	The adjectives do not indicate clearly if the resection is performed inside or outside the adventitial layer.
* **Sub-adventitial (cystectomy),** Adjective			The adjectives, with their definition, were rejected by a majority of voters (median: 0).
**Alveococcosis,** Noun	Disease related to infection with *E. multilocularis.*	Historical name for the infection due to *E. multilocularis* in Russia/Russian language and Russia-related countries.	Not in use in other countries/languages than Russia/Russian
		Not compliant with the recommendations of the World Federation of Parasitologists (WFP), since it is built from the name of a wrong genus.	The only recommended name is “Alveolar echinococcosis (AE)” (see Table 1C).
* **Anti-infectious,** * **Anti-infective, (therapy/treatment/drug),**	Drug treatment (usually chemical) of echinococcosis, opposed to or associated with surgery (on the model of infectious diseases therapy).	Although they are commonly used for the treatment of infectious diseases, these adjectives infer prevention as well as (and even rather than) treatment.“Anti-parasitic” is more exact, and more appropriate for parasitic diseases.	The adjectives “anti-infectious” and “anti-infective” were rejected by all voters (median: 10).
**Chemotherapy,** Noun	Drug treatment (usually chemical) of echinococcosis, opposed to or associated with surgery (on the model of cancer therapy)	The word was used at the first trials of mebendazole/albendazole, for the treatment of echinococcosis, in the 1980s, to stress the “chemical” nature of the treatment and oppose this treatment to surgery which was the only therapeutic option. Has been used commonly thereafter.	Even though echinococcosis shares some particularities with cancer, the word “chemotherapy” has gained a strong cancer-related meaning which may cause confusion, especially when echinococcosis occurs in cancer patients because of the immunosuppressive effect of the anti-cancer chemotherapy. In addition, not all drugs against echinococcosis are chemical (cf. immunotherapy and other biotherapies). It should be abandoned for the use of “anti-parasitic” therapy (or treatment, or drugs). See also Table 1C.
* **Closed cystectomy,** Expression	Surgical operation which does not include cyst opening before cyst removal (cystectomy).	Widely used by surgeons; however, linguistically incorrect: a cystectomy – which is an intervention – cannot be “closed”, and a cyst is rather “non-opened” than “closed” (the surgeon does not close the cyst, he/she may have it non-opened) [81].	The majority of voters rejected the expression (median: 3).The expression should be replaced by “non-opened cyst (NOC) -cystectomy”.
	Only applies to CE, and for total cystectomy; it is not relevant for AE.		See also Table 1C and Figure 3 and the description of the AORC framework with the definition of its various components in liver CE surgery.
* **Daughter hydatid,** Expression	Newly formed hydatids inside (and far less frequently outside, if any) the CE cyst in the development of *Echinococcus granulosus s.l.* Not applicable to *E. multilocularis*/AE.	Alternative term to “daughter cyst”. Although it fits well with the description of the “hydatid”, as a noun, it is not established by usage.	The term with this definition was rejected by the majority of voters (median: 1).
* **Daughter vesicle,** Expression	Newly formed hydatids inside (and far less frequently outside, if any) the CE cyst in the development of *Echinococcus granulosus s.l.* Not applicable to *E. multilocularis*/AE.	Alternative term to “daughter cyst”. As a literal translation, the use of “vesicle” would fit with the initial description by Dévé (“*vésicule fille*” in French).	The majority of voters rejected all suggested names for this metacestode structure (median: 3; mean: 4.7, for “daughter vesicle”). The arguments which prevailed to reject “daughter vesicle” and eventually approve the historical expression “daughter cyst” was first the worldwide usage and second the agreement by the participants in the subgroup “Biology and immunology” to restrict the use of “vesicle” to the metacestode of *Echinococcus* spp. *in vitro* only, whatever the species.
***Echinococcus alveolaris*,** Expression	Disease related to infection with *E. multilocularis.*	Name for the infection due to *E. multilocularis* sometimes used in German-speaking countries.	Mix of the name of the genus and the adjective qualifying the disease (in Latin…).
		Not compliant with the recommendations of the WFP.	The only recommended name is “Alveolar echinococcosis (AE)” (see Table 1C).
***Echinococcus cysticus,*** Expression	Disease related to infection with *E. granulosus sensu lato* (*s.l.*).	Name for the infection due to *E. granulosus s.l.* sometimes used in German-speaking countries	Mix of the name of the genus and the adjective qualifying the disease (in Latin…)
		Not compliant with the recommendations of the WFP.	The only recommended name is “Cystic echinococcosis (CE)” (see Table 1C).
**Hydatic,** Adjective	Related to *Echinococcus* spp.	Gallicism for “hydatid”, used as an adjective (“*hydatique*”, in French).	Should not be used in English.
**Hydatid disease,** Expression	Disease related to infection with *Echinococcus* spp.	Commonly used alternative name to designate either all diseases due to *Echinococcus* spp. or the diseases due to *E. granulosus sensu lato*. This disease name does not fit with the unified recommendations of the WFP.	This noun should not, in any cases, be used for alveolar echinococcosis or neotropical echinococcosis; it should not be used for *E. granulosus s.l.* infection in humans either: the only recommended name is “Cystic echinococcosis (CE)” (see Table 1C).
		In addition, usage of this name increases confusion among clinicians and decision makers between the diseases due to *E. granulosus sensu lato* and *E. multilocularis*, respectively.	Use of “hydatid” as an adjective should be restricted to infection due to *E. granulosus s.l.* (see Tables 1B and 1C) in the intermediate hosts.
**Hydatid polycystosis,** Expression	Cystic (or other) echinococcoses with multiple cysts	Sometimes used in publications. Source of confusion with polycystic (non-parasitic) diseases (see “polycystic”).	The adjective “multi-cystic” is now proposed as a non-specific description of any type of cystic echinococcosis when several/many macroscopic cysts are present and visible at imaging (see Table 1C).
			Use of “hydatid” as an adjective should be restricted to infection due to *E. granulosus s.l.* (see Table 1C) in the intermediate hosts.
* **Hydatidectomy,** Noun	Partial cystectomy, including the removal of the germinal and laminated layers of *E. granulosus sensu lato* cysts after cyst opening	From the definition of “hydatid” (see Table 1B), this noun could replace “partial cystectomy”, with less ambiguity (especially regarding surgeon’s evaluation between “subtotal” and “partial”).	Scientifically correct, but not established by use.
			The term with this definition was rejected by the majority of voters (median: 0).
**Hydatidosis,** Noun	Disease related to infection with *Echinococcus* spp.	Commonly used name to designate the diseases due to *Echinococcus* spp. This disease name does not fit with the recommendations of the WFP	This noun should not, in any cases, be used for alveolar echinococcosis or neotropical echinococcosis; it should not be used for *E. granulosus s.l.* infection in humans either: the only recommended name is “Cystic echinococcosis (CE)” (see Table 1C).
		In addition, usage of this name increases confusion among clinicians and decision makers between the diseases due to *E. granulosus s.l.* and *E. multilocularis*, respectively.	
* **Open cystectomy,** Expression	Surgical operation which includes cyst opening before cyst removal (cystectomy).	Expression widely used by surgeons; however, linguistically incorrect: a cystectomy cannot be “open”, but a cyst may be “opened” (the surgeon does open the cyst) [81].	The majority of voters rejected the expression (median: 3). The expression should be replaced by “non-opened cyst cystectomy” (NOC).
	Only applies to CE; may be at first or second intent; it is not relevant for AE.		See also Table 1C and Figure 3 and the description of the AORC framework with the definition of its various components in liver CE surgery.
**Pericystectomy**	Removal of a CE cyst which includes all layers of the cyst (including the adventitial layer).	Wrongly used to designate the operation which includes the adventitial layer of the cyst in CE. The prefix “peri” is not useful, since the adventitial layer is part of the cyst; “cystectomy” is thus the appropriate word. *Stricto sensu*, the “pericyst” is the normal organ parenchyma that surrounds the cyst (see Figs. 2A–2C) [68].	See Table 1C for alternative wording regarding surgery of CE.
			The only noun recommended is “cystectomy”.
			See also Figure 3 and the description of the AORC framework with the definition of its various components in liver CE surgery.
**Polycystic echinococcosis,** Expression	Disease related to infection with *E. vogeli* (stressing the multicystic type/presentation of *E. vogeli* infection).	The expression is sometimes wrongly used in medical publication to designate diseases due both to *E. vogeli*, which is most often polycystic/multicystic and *E. oligarthra,* which usually presents with single cysts similar to those of CE, in humans and in animal intermediate hosts [11, 35] but may also be multicystic or even microcystic (AE-like) [69].	The only recommended name for the diseases due to *E. vogeli* and *E. oligarthra* is “Neotropical echinococcosis (NE)” (see Table 1C).
		In addition, “polycystic” may cause confusion between the disease due to *E. vogeli* and “polycystic” clinical types of *E. granulosus* and *E. multilocularis* infections. The adjective “polycystic” is widely used to designate a genetic non-parasitic disease of the liver and kidney, adding to the confusion.	The adjective “multicystic” is now proposed as a non-specific description of any type of cystic echinococcosis when several/many macroscopic cysts are present and visible at imaging (see Table 1C).
**Polycystic,** Adjective	Constituted of several cysts (used to qualify the imaging and operative aspect of various types of echinococcosis).	The adjective “polycystic” is not specific to echinococcosis; “polycystic” is widely used to qualify a genetic non-parasitic disease of the liver and kidney; in addition, it has been used to distinctly qualify the disease due to *E. vogeli* (see below).	The adjective “multicystic” is now proposed as a non-specific description of any type of cystic echinococcosis when several/many macroscopic cysts are present and visible at imaging (see Table 1C).
**Protoscolicide*,*** Noun; **Protoscolicidal,** Adjective	Compound (natural or chemical) which is able to kill the protoscolex.	Synonym for protoscolecide. The protoscolex is the final stage of the metacestode of *Echinococcus* spp, able to produce a scolex then an adult form of the parasite in the definitive host, or to reproduce a metacestode in the intermediate host. From the ancient Greek “σκὠλεξ”-scolex (worm), genitive: σκὠλεκος-scolecos, and not scolicos; plural σκὠλεκες, scoleces, and not scolices), with the prefix “πρῶτος”-protos (first/before), and the Latin suffix –“-cide”, from “*caedere*” (to kill).	Should be abandoned for “protoscolecide” (see Table 1C); protoscolecides (protoscolecidal agents) are used to kill the protoscoleces and/or prevent secondary cysts after surgery.
** **Relapse**, Noun	Any type of echinococcosis lesions that appeared after the implementation of any type of treatment.	The definition is vague and covers several types of lesion appearing after attempts of treatment. More precise definitions of lesions observed in this situation should be used.	Should be abandoned for specific terms/expressions covering the various situations encountered by clinicians (see in Table 1C the definitions of “new CE cyst”, “reactivation”, “recurrence”, and “secondary echinococcosis”).
**Scolecide*,*** Noun; **Scolecidal,** Adjective	Compound (natural or chemical) that is able to kill the scolex.	The scolex is the head of the adult form of *Echinococcus* spp. From the ancient Greek “σκὠλεξ”, genitive: σκὠλεκος-scolecos; plural ςσκὠλεκες, scoleces). Such compounds are used to kill protoscoleces, produced by the metacestode in intermediate hosts, not scoleces in the definitive hosts. Usage of “scolecide” is thus scientifically inexact (killing concerns the protoscoleces, not the scoleces).	Should be abandoned for “protoscolecide” (see Table 1C).
**Scolicide*,*** Noun; **Scolicidal,** Adjective	Compound (natural or chemical) that is able to kill the scolex.	Synonym for scolecide. The common usage of “scolicide” is both scientifically inexact (killing concerns the protoscoleces, not the scoleces) and etymologically wrong (see also “scolecide” and “protoscolicide”).	Should be abandoned for “protoscolecide/protoscolecidal” (see Table 1C).

* Agreement to reject the term obtained at the second stage of the consultation (the participants in the Consultation and Rating Group had to rate their approval or rejection of the words/expressions independently on a scale from 0 to 10; whenever relevant, the median of the votes is given in the “Comments” column).

** Agreement to reject a term that was not selected at the first stage of consultation; the agreement was obtained at the second stage after further discussion between experts or after recommendation by the RRG.

#### Poll on debated issues

**Figure 3:**
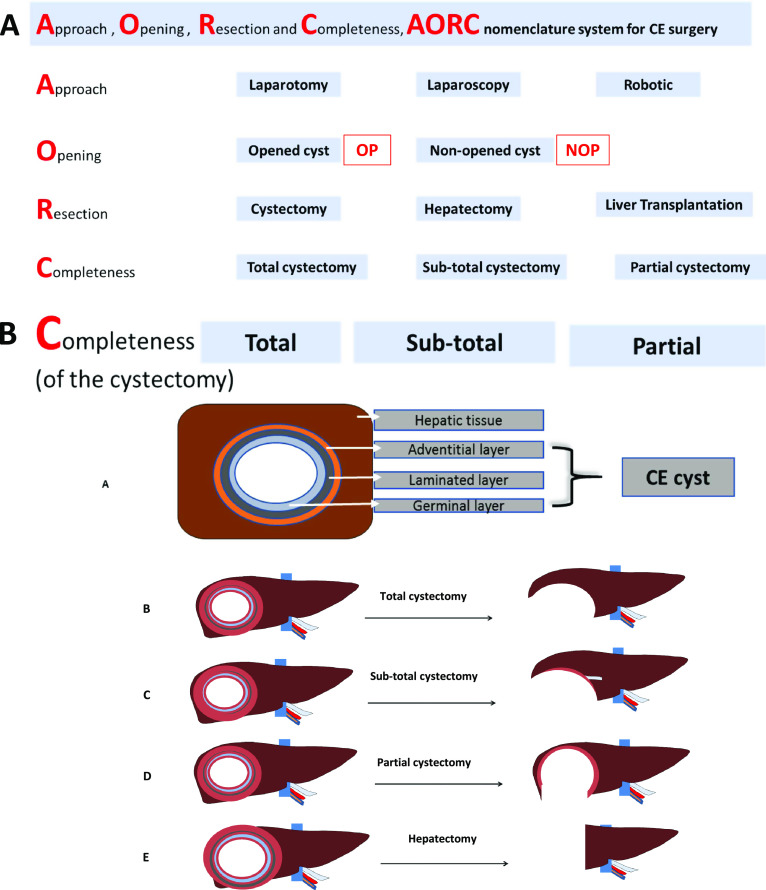
“AORC” nomenclature framework for CE surgery. (A) **(top)** – Vocabulary and acronyms. **AORC**: acronym for **A**pproach, **O**pening, **R**esection and **C**ompleteness; **CE**: cystic echinococcosis; **OC**: opened cyst; **NOC**: non-opened cyst. (B) **(bottom)** – A. Schematic structure of CE cyst: the CE cyst consists (from inside to outside) of the germinal layer, laminated layer and adventitial layer. B. Total cystectomy requires the resection of all three layers completely. C. Subtotal cystectomy requires the nearly total resection of all three layers; only parts of the adventitial layer are preserved because of surgical safety. D. Partial cystectomy refers to the incomplete resection of any of three layers (usually of the adventitial layer) due to technical and safety issue. E. Hepatectomy requires the *en bloc* resection of part of liver parenchyma, following the rules of hepatic resection.

Among the 39 final participations in the poll about “debated issues” nine were incomplete; the questionnaire was sent back to these participants for completion; precise answers were eventually obtained. The ratings provided for the poll on “debated issues” were analyzed in order to obtain means and medians, and thus a final score for approval or rejection of each term. Results of the median rating for the “debated terms”, and whenever useful the agreement between participants, are given in the “Comments” column of Tables 1A–1C and 2A–2C. Objections and concerns on the use of some terms were re-discussed by specialists before making a final decision on their final rejection or approval, and minor modifications in the wording of definitions were made in order to obtain a final consensus. An important result of the poll, since it was distinctly and clearly agreed upon both by the participants in the “Biology and immunology” subgroup and those in the “Clinical aspects” subgroup, was on the word “hydatid”. According to the polls, all names of diseases or operations composed from the radical “hydatid” (e.g., in “hydatid-osis” or “hydatid-ectomy”, etc.) should be abandoned, and the adjective “hydatid”, whenever used, should never be used to qualify entities different from those due to *E. granulosus sensu lato*, thus never be used for *E. multilocularis*, *E. vogeli* and *E. oligarthra*. A final discussion on the characterization of the surgical procedures aimed at removing CE cysts eventually retained a simple framework that should be used whenever surgical operations are described in publications, with four levels of mandatory qualification, regarding (1) **A**pproach: “laparotomy”, “laparoscopy” or “robotic”; (2) Cyst **O**pening: “non-opened cyst” (NOP) versus “opened-cyst” (OP); (3) **R**esection type: “cystectomy”, “hepatectomy”, or “liver transplantation”; and (4) **C**ompleteness of resection: “total”, “subtotal”, and “partial”. Description of the **AORC** unified framework for surgical interventions in CE is given in Figures 3A and 3B. Precise description of the procedure actually performed should complete the mandatory terms (description of the removed parasite layers, area in square centimetres or percentage of cyst actually resected, closure of communications with bile ducts, etc.).

#### Review by the RRG and meeting at the 28th WCE in Lima, Peru

Feedback from the RRG members usually addressed minor aspects of text readability and/or definitions/descriptions in the Tables. Clarification was provided by the reviewers on the definition of the species and their hosts, especially regarding species involved in NE. The major issues raised by one reviewer (clinician) concerned the definition of the “cyst”, and the retention of the adjective “hydatid”, even in its very restrictive sense as described in Tables 1B and 1C. However, because of the clear results of the consensus process, these objections could not be taken into account in the final version. At the meeting of the WHO-IWGE held in Lima, Peru, during the 28th WCE, the still debated issues were raised and final adjustments were made between the SWG and the writers of the WHO-IWGE Technical Manual on CE treatment. Words and expressions finally approved and rejected after the poll and the last discussions were added to the list of approved and rejected terms. Terms finally approved (95 items) are thus included in Tables 1A (Genetics and epidemiology), 1B (Biology and immunology) and 1C (Clinical aspects). Rejected terms (60 items) are included in Tables 2A, (Species and epidemiology), 2B (Biology and immunology) and 2C (Clinical aspects). To facilitate consultation, Tables 1A–1C and 2A–2C are organized by topics of interest (A, B and C), and within each topic, in alphabetic order of the words or expressions.

## Discussion

This 2-year multidisciplinary and international exchange of views has allowed physicians, veterinarians and basic science researchers to freely discuss opinions about terms used in all fields relating to *Echinococcus* spp., and to reach a consensus on the use of the most appropriate ones, based on sound scientific and/or linguistic grounds. When evidence was not available, consensus was based on reasoned arguments, including usage when no suitable alternative was found, confirmed by a poll. This is the first and only endeavour of this type in the domain of echinococcosis; and it is endorsed by the WAE. Definitions of 155 terms and expressions and recommendations provided in Tables 1A–1C and 2A–2C and Figures 2A–2C and 3 should be followed in the future by all scientists writing scientific papers in English; scientific editors and referees/reviewers should recommend article authors to use the approved terms. These recommendations should help the recognized experts, involved in scientific work on echinococcosis at the international level, to set up a similar list of recommended and rejected words in their country language(s), so that similar wording could be used by all professionals. Major advances promoted by the multidisciplinary work on echinococcosis terminology were: (1) the confirmation of the current recognition of nine species of *Echinococcus*; (2) an agreement on the names of the three types of diseases due to *Echinococcus* spp. and rejection of all others; (3) the restriction of the adjective “hydatid” to *E. granulosus s.l.*; and (4) the proposal of the “AORC” framework to describe surgical interventions in CE.

The participants involved in the work were representative of the “echinococcosis” scientific community. Half of the 61 participants in the three working groups belonged to the top 50 experts in the area (as cited from their publications retrieved with the key words “echinococcosis”: http://expertscape.com/ex/echinococcosis or “hepatic echinococcosis”: http://expertscape.com/ex/echinococcosis%2C+hepatic). Health professionals and young researchers were also involved in the CRG; there was a balanced proportion of researchers and professionals. The participants in the three groups were from 15 endemic countries, and all five continents were represented with a balance of male and female contributors to the debate. Recruitment of the participants in the CRG at the 27th WCE on a volunteer basis enabled researchers and professionals from the Maghreb and Middle East countries, highly endemic countries for CE, to participate in the CRG. Additional colleagues, especially from South America and China, were solicited after the congress. However, only Chinese colleagues answered positively, which resulted in a relatively low participation of South American colleagues in the “Clinical aspects” subgroup, perhaps because of language issues: Spanish and Portuguese are the publication languages of most South American clinicians; publications in English from South America are mostly in biology and public health. All members of the RRG were selected by the SWG on the basis of their international academic recognition in the fields of CE, AE and NE. Despite the absence of face-to-face meetings to discuss the undecided questions, open email discussions were initiated by the coordinators of the subgroups, and this procedure was very efficient. If there were missing values for the poll, the project manager actively and individually contacted the voters in question in order to ask them to provide their opinion. Feedback from the RRG members was also followed by active discussions in order to reach consensus on important terms and to revise and finalize this paper.

### Major outcomes and conclusions

Currently, a precise genetic definition is available for nine species: *E. granulosus* (Batsch, 1786) *sensu stricto*, *E. canadensis* (Webster & Cameron, 1961), *E. ortleppi* Lopez-Neyra & Soler Planas, 1943, *E. felidis* Ortlepp, 1937, and *E. equinus* (Williams & Sweatman, 1963), within the *E. granulosus* (Batsch, 1786) *sensu lato* cluster and responsible for cystic echinococcosis, *E. multilocularis* (Leuckart, 1863), and *E. shiquicus* Xiao, Qiu, Nakao, Li, Yang, Chen, Schantz, Craig & Ito, 2005, responsible for alveolar echinococcosis, and *E. vogeli* Rausch and Bernstein, 1972 and *E. oligarthra* (Diesing, 1863), responsible for neotropical echinococcosis; the responsibility of some of the species (e.g., *E. equinus* (Williams and Sweatman, 1963) or *E. shiquicus* Xiao et al., 2005) in human infection is still being questioned; this does not challenge the genetic definition of these species (Table 1A). Recognition of additional species within *E. canadensis* and *E. granulosus s.s.* is to be expected (Table 1A). Thus, distinct *Echinococcus* spp. genotypes within the *E. canadensis* cluster may move to full species status, and the names *E. intermedius* (for G6/7), *E. borealis* (for G8) and *E canadensis* (for G10) have been proposed; however, no conclusion on the number and names of these species has yet been reached [57].

Variations around the radical “hydatid” have long been used to designate diseases due to *Echinococcus* spp. The presence of “hydatid” cysts had long been recognized as a feature of a parasitic disease when the natural cycle of the parasite responsible for its occurrence was identified in the middle of the 19th century [18, 77]; the species was first named *Taenia echinococcus* before becoming *Echinococcus granulosus*. In humans, the disease was clearly not a “taeniasis”, and “hydatid disease”, “hydatidosis” (if the disease was considered) or “hydatid cyst” (if the lesion was considered) thus prevailed. It took another century before the *Echinococcus* sp. which causes AE was identified as a distinct species, *E. multilocularis* [18, 77]. It is thus easy to understand why several terms, not related to the current name of the genus, were used for centuries, and why they were used to designate both diseases, alveolar and cystic echinococcosis. The inappropriate use of “hydatid” has been a source of confusion, both for disease surveillance and treatment indication [10, 35]. Until recently, echinococcosis surveillance by the European Centre for Disease Control (ECDC) did not distinguish between the two diseases [23]. A clear distinction between three types of diseases, with a single name for each of them, will make all studies on epidemiology, socio-economic burden, and care management more reliable. There was total agreement to never use terms based on the “hydatid” root to designate any disease due to *Echinococcus* spp., and to strictly restrict the use of “hydatid” to qualify a lesion or part of a lesion due to *E. granulosus s.l.* in intermediate hosts, entirely excluding its use to qualify anything regarding (1) *E. granulosus s.l.* infection of the definitive host, and (2) AE and NE. With regards to a cystic lesion, “CE cyst” should be preferred to “hydatid cyst” since the large majority of voters preferred to use this term, and did not wish to keep both names for *E. granulosus s.l*.-related cysts.

Although specialists of the genetics of *Echinococcus* spp. rapidly agreed on the correct spelling of the species *E. oligarthra* on historical and linguistic arguments (and not *oligarthrus*, as commonly spelled in the last few decades; see e.g., [5, 17]), clinicians were more hesitant on the names of the diseases that *E. oligarthra* and *E. vogeli* may cause in humans. There was no term/expression entirely satisfactory to designate both diseases. Because both are infections of the “tropical areas of the New World”, “neotropical” has been commonly used in the scientific literature and was presented at the WHO-Forum in 2015 to be included among the U50-U99 “Codes for research and alternative subclassifications” of the International Statistical Code of Diseases and Related Health Problems (https://apps.who.int/iris/bitstream/handle/10665/246208/9789241549165-V2-eng.pdf), as: U51X: “Infection due to Neotropical Echinococcosis”. The suggestion of South American specialists, which was confirmed by the poll, was thus to keep this denomination. To avoid the confusion with “polycystic liver and kidney disease”, a genetic disease, the adjective “polycystic” (of Greek origin) should definitively be replaced by “multicystic” (of Latin origin, but with exactly the same meaning) when multiple aggregated cysts are observed on imaging, regardless of the species; “multiple cyst-” characterizes the co-existence of several separated and independent cysts in the same organ or different organs.

It was agreed that the current international classifications already approved by the WHO-IWGE, i.e. the “CE” classification of CE cysts [82], and the PNM classification of AE lesions [37], would not be questioned, and that further work on the refinement of imaging classification of AE would not proceed until fixing of international recommendations. However, an unexpected and extremely useful outcome of the terminology discussions was the agreement on a new and internationally recognized system to describe surgical operations in CE, the “AORC” framework (Fig. 3), with consensus on the single word “cystectomy” to describe the removal of the CE cyst, thus excluding “pericystectomy” when it consists of the removal of the three layers of the cyst without “pericystic” liver. This recommendation will help professionals to share a common description that is relevant in terms of perioperative risks and the risk of CE recurrence after operation. A precise description of the procedure actually performed by the surgeon for a given patient should accompany the mandatory terms (description of the removed parasite layers, area in square centimeters or percentage of cyst actually resected, closure of communications with bile ducts, content of the cyst if it was opened, etc.); this is beyond the objectives of a work on terminology and will be further discussed in the “Technical Manual” for the diagnosis and treatment of CE, which is currently being prepared by the WHO-IWGE. For non-surgical CE interventions, the clear distinction between the PAIR (puncture, aspiration, injection, reaspiration, through a needle, without catheterization), S-CAT (standard catheterization), and Mo-CAT (modified catheterization) techniques provides accepted acronyms and will also help professionals to better understand the specific indications of each technique and the results of clinical trials.

The work in terminology aimed to cover all fields of echinococcosis; it was the first attempt at standardizing scientific and medical language in a specific area of parasitic disease – echinococcosis. However, it did not claim to solve all points of debate and/or to address everything definitively. The words and expressions reported in Tables 1A–1C and 2A–2C do not encompass all technical aspects; such details should be considered by subgroups of specialists. Regular updating will also be necessary, following future advances in scientific and medical knowledge. Apparently “easy-to-solve” dilemmas were in fact more difficult to resolve when several disciplines reported on their own usages. This was the case for the debate between “daughter cyst” and “daughter vesicle”. Purists will certainly regret that “daughter cyst” will stay in use, despite the paradoxical female gender, explained by the use of the feminine “*vésicule*” (vesicle) in the first descriptions in French. Although “daughter” is not justified in English, the alternative adjective “secondary” cannot replace it since it is widely used to designate new cysts developed in the peritoneum, pleura or meningeal space after cyst rupture and/or protoscolex spillage Although “cyst” was not fully justified either, because it has no fully developed adventitial layer, biologists readily agreed to keep the noun “vesicle” with restricted use for the *in vitro* situation. The results of the poll (Tables 1C and 2C) confirmed the problematic nature of the issue: “daughter cyst” was not preferred, “daughter vesicle” was not either, and the alternative expression “daughter hydatid” was unequivocally rejected; it was thus agreed to keep the “historical wording”, i.e. “daughter cyst”. We recommend the constitution of a permanent group on “terminology” common to the WAE and the WHO-IWGE so that such “unresolved” issues may be further discussed, and that the certainly numerous new terms used in the field in the future may be handled in a timely manner.

## Appendix

### List of the 9 participants in the Steering and Writing Group (SWG)

Project manager: Vuitton Dominique A.

#### Subgroup coordinators:

*Species and epidemiology*: McManus Donald P., Australia; Romig Thomas, Germany.

*Biology and immunology*: Rogan Michael R., UK; Gottstein Bruno, Switzerland.

*Clinical aspects*: Menezes da Silva Antonio*, Portugal; Wen Hao, PR China.

*Data collection and writing assistants*: Naidich Ariel, Argentina; Tuxun Tuerhongjiang, PR China.

**Representative of the World Association of Echinococcosis*, as Past-President.

### List of the 42 participants in the Consultation and Rating Group (CRG)

#### Subgroup “Species and epidemiology” (15 participants, listed in alphabetic order)

Avcioglu Amza, Turkey; Boufana Belgees, UK; Budke Christine, USA; Casulli Adriano, Italy; Güven Esin, Turkey; Hillenbrand Andreas, Germany; Jalousian Fateme, Iran; Jemli Mohamed Habib, Tunisia; Knapp Jenny, France; Laatamna Abdelkarim, Algeria; Lahmar Samia, Tunisia; Naidich Ariel, Argentina; Rogan Michael T., UK; Sadjjadi Seyed Mahmoud, Iran; Schmidberger Julian, Germany.

#### Subgroup “Biology and immunology” (15 participants, listed in alphabetic order)

Amri Manel*, Algeria; Bellanger Anne-Pauline, France; Benazzouz Sara*, Algeria; Brehm Klaus, Germany; Hillenbrand Andreas, Germany; Jalousian Fateme, Iran; Kachani Malika, USA/Morocco; *Labsi Moussa, Algeria; Masala Giovanna, Italy; Menezes da Silva Antonio, Portugal; Sadjjadi Seyed Mahmoud, Iran; Soufli Imene*, Algeria; Touil-Boukoffa Chafia*, Algeria; Wang Junhua, Switzerland/PR China; Zeyhle Eberhard, Kenya.

#### Subgroup “Clinical aspects” (12 participants, listed in alphabetic order)

Aji Tuerganaili, PR China; Akhan Okan, Turkey; Bresson-Hadni Solange, France; Dziri Chadli, Tunisia; *Gräter Tilmann, Germany; *Grüner Beate, Germany; Haïf Assia, Algeria; Hillenbrand Andreas, Germany; Koch Stéphane, France; Rogan Michael T., UK; Tamarozzi Francesca, Italy; Tuxun Tuerhongjiang, PR China.

** Rating from these participants, who worked together, was considered as only one rating.*

### List of the 12 participants in the Reading and Review Group (RRG)

#### Subgroup “Species and epidemiology” (4 participants, listed in alphabetic order):

Giraudoux Patrick, France; Torgerson Paul, Switzerland/Central Asia; Vizcaychipi Katherina, Argentina; Xiao Ning, PR China.

#### Subgroup “Biology and immunology” (4 participants, listed in alphabetic order):

Altintas Nazmiye, Turkey; Lin Renyong, PR China; Millon Laurence, France; Zhang Wenbao, PR China.

#### Subgroup “Clinical aspects” (4 participants, listed in alphabetic order):

Achour Karima, Algeria; Fan Haining, PR China; Junghanss Thomas, Germany; Mantion Georges A., France.

### Names, affiliations and email addresses of Collaborators

Algeria

**Achour Karima**, Service de chirurgie thoracique, cardio-vasculaire et de transplantation rénale, Hôpital universitaire Mustapha, DZ-16000 Bab-El-Oued, Algeria; achour.karima@gmail.com

**Amri Manel**, Laboratoire de Biologie Cellulaire et Moléculaire, Faculté des Sciences Biologiques, Université des Sciences et Technologie Houari Boumediene, DZ-16111 Algiers, Algeria; manelamri@yahoo.fr

**Benazzouz Sara**, Laboratoire de Biologie Cellulaire et Moléculaire, Faculté des Sciences Biologiques, Université des Sciences et Technologie Houari Boumediene, DZ-16111 Algiers, Algeria; sara.and1@hotmail.fr

**Haïf Assia**, Service de Chirurgie Pédiatrique, Université Ferhat Abbas, DZ-19000 Setif, Algeria; haif_chu.setif@yahoo.fr

**Laatamna Abdelkarim**, Département de Parasitologie, Université Ziane Achour, DZ-B.P. 3117 Djelfa, Algeria; laatamnaabdelkarim@yahoo.com

**Labsi Moussa**, Laboratoire de Biologie Cellulaire et Moléculaire, Faculté des Sciences Biologiques, Université des Sciences et Technologie Houari Boumediene, DZ-16111 Algiers, Algeria; touilboukoffa@yahoo.fr

**Soufli Imene**, Laboratoire de Biologie Cellulaire et Moléculaire, Faculté des Sciences Biologiques, Université des Sciences et Technologie Houari Boumediene, DZ-16111 Algiers, Algeria; touilboukoffa@yahoo.fr

**Touil Boukoffa Chafia**, Laboratoire de Biologie Cellulaire et Moléculaire, Faculté des Sciences Biologiques, Université des Sciences et Technologie Houari Boumediene, DZ-16111 Algiers, Algeria; touilboukoffa@yahoo.fr

Argentina

**Naidich Ariel,** Departamento de Parasitología, Instituto Nacional de Enfermedades Infecciosas, ANLIS “Dr. Carlos G. Malbrán”, AR-CP 1281 - Buenos Aires, Argentina; anaidich@anlis.gob.ar

**Vizcaychipi Katherina**, Instituto Nacional de Medicina Tropical (INMeT) Pto. Iguazú Misiones, Ministerio de Salud de la Nación, AR- CP 1281 Buenos Aires, Argentina; kvizcaychipi@gmail.com

Australia

**McManus Donald P.**, Molecular Parasitology Laboratory, Infectious Diseases Division, QIMR Berghofer Medical Research Institute, AU-4006 Herston-Brisbane, QLD, Australia; don.McManus@qimrberghofer.edu.au

China

**Aji Tuerganaili**, Department of Hepatic Surgery; WHO Collaborating Centre for Prevention and Care Management of Echinococcosis; 1st Affiliated Hospital of Xinjiang Medical University, CN-830011 Urumqi, China; dr.wenhao@163.com

**Fan Haining**, China; Department of Hepatic Surgery; 1st Affiliated Hospital of Qinghai University, CN-810001 Xining, China; fanhaining@medmail.com.cn

**Lin Renyong**, State Key Laboratory of Pathogenesis, Prevention and Treatment of High Incidence Diseases in Central Asia, CN-830011 Urumqi, China; renyong_lin@sina.com

**Tuxun Tuerhongjiang**, Department of Hepatic Surgery; WHO Collaborating Centre for Prevention and Care Management of Echinococcosis; 1st Affiliated Hospital of Xinjiang Medical University, CN-830011 Urumqi, China; turgunbay@163.com

**Wen Hao**, WHO Collaborating Centre for Prevention and Care Management of Echinococcosis & State Key Laboratory of Pathogenesis, Prevention and Treatment of High Incidence Diseases in Central Asia, CN-830011 Urumqi, China; dr.wenhao@163.com

**Xiao Ning**, National Institute of Parasitic Diseases, Chinese Centers for Disease Control, CN-200025 Shanghai, China; ningxiao116@126.com

**Zhang** Wenbao, WHO Collaborating Centre for Prevention and Care Management of Echinococcosis & State Key Laboratory of Pathogenesis, Prevention and Treatment of High Incidence Diseases in Central Asia, CN-830011 Urumqi, China; wenbaozhang2013@163.com

France

**Bellanger Anne-Pauline**, Laboratoire de Parasitologie et Mycologie; Centre National de Référence pour les Echinococcoses, Centre Hospitalier Régional Universitaire, FR-25030 Besançon, France; apbellanger@chu-besancon.fr

**Bresson-Hadni Solange**, Centre National de Référence pour les Echinococcoses, Centre Hospitalier Régional Universitaire, FR-25030 Besançon, France; solange.bresson.sbh@gmail.com

**Giraudoux Patrick**, UMR Chrono-environnement; Université Bourgogne Franche-Comté, FR-25030 Besançon, France; patrick.giraudoux@univ-fcomte.fr

**Knapp Jenny**, France; Laboratoire de Parasitologie; Centre National de Référence pour les Echinococcoses, Centre Hospitalier Régional Universitaire, FR-25030 Besançon, France

**Koch Stéphane**, Service de Gastroentérologie; Centre Hospitalier Régional Universitaire, FR-25030 Besançon, France; skoch@chu-besancon.fr

**Mantion Georges**, Université Bourgogne Franche-Comté, FR-25030 Besançon, France; gmantion@univ-fcomte.fr

**Millon Laurence**, Laboratoire de Parasitologie; Centre National de Référence pour les Echinococcoses, Centre Hospitalier Régional Universitaire, FR-25030 Besançon, France; lmillon@univ-fcomte.fr

**Vuitton Dominique Angèle**, EA 3181, Université Bourgogne Franche-Comté, FR-25030 Besançon, France; dvuitton@univ-fcomte.fr

Germany

**Brehm Klaus**, Institut für Hygiene und Mikrobiologie Universität Würzburg, DE-97080 Würzburg; Germany; kbrehm@hygiene.uni-wuerzburg.de

**Gräter Tilmann**, Klinik für Diagnostische und Interventionelle Radiologie Universitätsklinikum Ulm, DE-89081 Ulm; Germany; tilmann.graeter@uniklinik-ulm.de

**Grüner Beate**, Comprehensive Infectious Diseases Center; Klinik für Innere Medizin III, Universitätsklinikum Ulm, DE-89081 Ulm, Germany; beate.gruener@uniklinik-ulm.de

**Hillenbrand Andreas**, Klinik für Allgemein- und Viszeralchirurgie, Universitätsklinikum Ulm, DE-89081 Ulm, Germany; andreas.hillenbrand@uniklinik-ulm.de

**Junghanss Thomas**, Sektion Klinische Tropenmedizin, Universitätsklinikum Heidelberg, Heidelberg, DE-69120 Germany; thomas.junghanss@urz.uni-heidelberg.de

**Romig Thomas**, Department of Parasitology, Fachgebiete Parasitologie, Universität Hohenheim, D-70593 Stuttgart, DE-70599 Stuttgart, Germany; thomas.romig@uni-hohenheim.de

**Schmidberger Julian**, Klinik für Innere Medizin I, Universitätsklinikum Ulm, DE-89081 Ulm, Germany; julian.schmidberger@uniklinik-ulm.de

Italy

**Casulli Adriano**, WHO Collaborating Centre for the Epidemiology, Detection and Control of Cystic and Alveolar Echinococcosis; European Union Reference Laboratory for Parasites; Foodborne and Neglected Parasitic Diseases Unit, Department of Infectious Diseases, Istituto Superiore di Sanità, IT-00161 Rome, Italy; adriano.casulli@iss.it

**Masala Giovanna**, Istituto Zooprofilattico Sperimentale della Sardegna, IT-07100 Sassari, Italy; giovanna.masala@izs-sardegna.it

**Tamarozzi Francesca**, WHO Collaborating Centre for the Epidemiology, Detection and Control of Cystic and Alveolar Echinococcosis; Department on Infectious Diseases, Istituto Superiore di Sanità, IT-00161 Rome, Italy; f_tamarozzi@yahoo.com

Iran

**Jalousian Fateme**, Department of Parasitology, Faculty of Veterinary Medicine, University of Tehran, IR-1417466191Iran; jalousian_f@ut.ac.ir

**Sadjjadi Seyed Mahmoud**, Dept. Parasitology and Mycology, School of Medicine, Shiraz University of Medical Sciences, IR-PO Box 71348-14336 Shiraz, Iran; sadjjadi316@gmail.com

Kenya

**Zeyhle Eberhard**, Meru University of Sciences and Technology, KE-60200 Meru, Kenya; zeana07@gmail.com

Morocco

**Kachani Malika**, Département de Parasitologie Institut Agronomique et Vétérinaire Hassan II, MA-BP 6202, Rabat, Morocco & College of Veterinary Medicine, Western University of Health Sciences, US-91766-1854 Pomona, CA, USA; mkachani@westernu.edu

Portugal

**Menezes da Silva Antonio**, World Association of Echinococcosis (Past-President); College of General Surgery of the Portuguese Medical Association (Associação dos Médicos Portugueses), PT-1749–084 Lisbon, Portugal; mensilvapt@yahoo.com

Switzerland

**Gottstein Bruno**, Institut für Parasitologie, Vetsuisse Facultät Bern, & Immuno-parasitologie, Medizinische Fakultät, Universität Bern, CH-3008 Bern, Switzerland; bruno.gottstein@vetsuisse.unibe.ch

**Torgerson Paul**, Abteilung für Epidemiologie, Vetsuisse Facultät Zürich, Universität Zürich, CH-8057 Switzerland; paul.torgerson@access.uzh.ch

**Wang Junhua**, Institut für Parasitologie, Vetsuisse Facultät Bern, Universität Bern, CH-3008 Bern, Switzerland; junhua.wang@vetsuisse.unibe.ch

Tunisia

**Dziri Chadli**, Département de Chirurgie Générale, Faculté de Médecine de Tunis, & Centre de Simulation Médicale Honoris, Université El Manar, TN-1068 Tunis, Tunisia; chadli.dziri@planet.tn

**Jemli Mohamed Habib**, Service de Parasitologie- Département Clinique de l’École Nationale de Médecine Vétérinaire – TN-2020 Sidi Thabet, Tunisia; jemli.medhabib@yahoo.fr

**Lahmar Samia**, Service de Parasitologie, Département Clinique de l’École Nationale de Médecine Vétérinaire, TN-2020 Sidi Thabet, Tunisia; drlsamia@yahoo.fr

Turkey

**Akhan Okan**, Department of Radiology, Hacettepe Üniversitesi/University, TR- 06230 Ankara, Turkey; akhano@tr.net

**Altintas Nazmiye**, Department of Parasitology, Ege Üniversitesi/University, TR-35040 Bornova-Izmir, Turkey; nazmiye.altintas@ege.edu.tr

**Avcioglu Amza**, Department of Veterinary Parasitology, Ataturk Üniversitesi/University, TR-25030 Yakutiye-Erzurum,Turkey; havcioglu@atauni.edu.tr

**Güven Esin**, Department of Parasitology, Ataturk Üniversitesi/University, TR-25030 Yakutiye-Erzurum, Turkey; esinguven@atauni.edu.tr

United Kingdom

**Boufana Belgees**, National Reference Laboratory for *Trichinella* & *Echinococcus*, National Wildlife Management Centre, Animal and Plant Health Agency, GB-YO41 1LZ Sand Hutton, York, United Kingdom; belgees.boufana@apha.gov.uk

**Rogan Michael**, School of Science, Engineering & Environment, University of Salford, GB-M5 4WT, Manchester, United Kingdom; m.t.rogan@salford.ac.uk

USA

**Budke Christine**, Department of Veterinary Integrative Biosciences, College of Veterinary Medicine & Biomedical Sciences, Texas A&M University, US-77843-4458 College Station, TX, USA; cbudke@cvm.tamu.edu
